# Effects of emotional content on social inhibition of gaze in live social and non-social situations

**DOI:** 10.1038/s41598-023-41154-w

**Published:** 2023-08-29

**Authors:** Laura Pasqualette, Louisa Kulke

**Affiliations:** 1 Department of Neurocognitive Developmental Psychology, Friedrich-Alexander-Universität Erlangen-Nürnberg, Erlangen, Germany; 2 Developmental Psychology with Educational Psychology, University of Bremen, Bremen, Germany

**Keywords:** Human behaviour, Psychology, Emotion, Social behaviour

## Abstract

**Abstract:**

In real-life interactions, it is crucial that humans adequately respond to others’ emotional expressions. Emotion perception so far has mainly been studied in highly controlled laboratory tasks. However, recent research suggests that attention and gaze behaviour significantly differ between watching a person on a controlled laboratory screen compared to in real world interactions. Therefore, the current study aimed to investigate effects of emotional expression on participants’ gaze in social and non-social situations. We compared looking behaviour towards a confederate showing positive, neutral or negative facial expressions between live social and non-social waiting room situations. Participants looked more often and longer to the confederate on the screen, than when physically present in the room. Expressions displayed by the confederate and individual traits (social anxiety and autistic traits) of participants did not reliably relate to gaze behaviour. Indications of covert attention also occurred more often and longer during the non-social, than during the social condition. Findings indicate that social norm is a strong factor modulating gaze behaviour in social contexts.

**Protocol registration:**

The stage 1 protocol for this Registered Report was accepted in principle on September 13, 2021. The protocol, as accepted by the journal, can be found at: 10.6084/m9.figshare.16628290.

## Introduction

Social interactions play a crucial role for humans and it is particularly important that people correctly identify others’ emotions during those exchanges to respond to them appropriately. However, attention to social stimuli has often been studied in non-social laboratory settings. The current study contributes to filling this gap by investigating effects of emotional expressions on attention in social and non-social situations.

Numerous laboratory studies show that socially relevant stimuli attract humans’ attention and gaze: Observers looking at photos of natural social and non-social scenes fixate first and more frequently at people, especially their eyes and head, than at other objects in the scene^1, 2^. In fact, eyes attract attention almost automatically^3, 4^. The automaticity of face processing is highlighted by research showing that normal faces are more easily detectable than inverted faces or scrambled faces, suggesting that there is a processing advantage for the particular arrangement of a human face^5–7^. Faces are also processed faster in the brain than words and objects^8^. The brain favoring the processing of social stimuli over others is in line with sociality’s important evolutionary role for the increase of the executive brain capacities, as suggested by the *social brain hypothesis*^9^. In summary, social stimuli automatically capture processing capacity and gaze.

Despite the significant role of social and emotional stimuli during every day interaction, most previous studies presented social stimuli on a computer screen in a controlled non-social laboratory environment. However, such impoverished settings lack information that only complex social environments offer about how visual information and social rules control where we look, and how we act^10, 11^. Most importantly, in controlled laboratory settings, gaze has the main function to *gather* information about the visual scene through looks at relevant stimuli. However, in a live social interaction, an additional function of gaze is to *signal* to others^12–14^. Argyle and Cook (1976) first mentioned this *dual function of gaze*, by stating, “Whenever organisms use vision, the eyes become signals as well as channels” (p. xi; Argyle & Cook, 1976)^12^. Therefore, humans’ gaze does not necessarily reflect their focus of attention or their belief-tracking^15^, as they can flexibly use the social function of gaze to follow implicit rules of when to look or not to look at others.

In social situations, but not in a laboratory context, human gaze patterns follow several implicit rules. For example, when unacquainted people enter the same space, they owe each other a quick gaze followed by withdrawal, which signals acknowledgement of the other person, but no desire to establish communication (theory of civil inattention)^16, 17^. If desirable, two strangers looking at each other can serve as a prompt to initiate and maintain conversation^18, 19^. On the other hand, when not desired, for instance when walking in public spaces, people tend of avoid following the gaze of others approaching towards them, since it increases the chance of social interaction^20^. Ellsworth and colleagues (1972) demonstrated that when participants are stared at, they tend to adopt a flight behavior and get away faster from the location than participants who were not stared at^21^. Summing up, even though people can use the gathering function when looking at stimuli on a computer screen, the signaling function is lost. This signaling function severely affects where people look, in line with implicit rules. It is therefore necessary to continue studying social phenomena in real life situations.

Recent studies have focused on investigating human gaze during real-life interactions to get a clearer picture of gaze in social situations. Although participants mostly look at people’s eyes when viewing videos or photos of people^22–24^, the same does not occur during social interactions in real life. Differences in gaze between live and non-live situations depend on the possibility of social interaction. For example, individuals often fixate other pedestrians that are not in close proximity (i.e. too far away to interact with), but avoid directly looking at them when they come closer. In contrast, when they watch non-live videos of the same recorded scenes, participants prefer to look at people close-by^25^. Laidlaw et al. (2011) performed a ground-breaking study, in which they compared gaze patterns of participants sitting in a waiting room. In one condition, a confederate was sitting in the waiting room with the participant, while in another condition a videotape of the confederate was displayed on a monitor. Interestingly, participants looked at the videotaped confederate considerably more often and overall longer, than when the confederate was physically present^26–28^. Although participants look less at a live confederate, this does not mean that they are uninterested in this other person. Pedestrians use covert attention (i.e. attention without direct looks) to decide if they should direct their gaze to another individual, chancing social interaction^29^. More recently, Dosso, Hyuhn, & Kingstone (2020) showed that participants pay more covert attention to a confederate who is physically present, but they overtly look at videos of the same confederate^30^. Similar neural mechanisms underlie overt and covert attention^31, 32^, involving enhanced processing of stimuli. However, participants may retain more top-down control during overt than covert shifts of attention^33^. Particularly, the suppression of gaze during covert shifts may require additional processing resources^31^. Therefore, as a social strategy, covert attention can be used to gather information about a target without offering social cues (e.g. perceiving people’s emotional states in several circumstances without staring at them). However, it may require inhibition of a prepotent overt attention shift and is therefore a less efficient way to visually explore and deeply process the object of interest.

As a specific social signal, emotional expressions attract attention. Emotion perception has evolutionary significance, as appropriate approach behavior (e.g. alimentation, reproduction, nurture of progeny) towards “appetitive” stimuli with a positive valence or avoidance of aversive stimuli (e.g. threat, danger), is crucial for survival^34, 35^. A bias to emotional compared to neutral faces has been demonstrated in human children and orangutans, indicating an ontogenetic and phylogenetic root^36^. Furthermore, emotion perception is crucial for successful social interactions, as atypical emotion processing can lead to severe impairments, for example in disorders such as autism spectrum disorder^37^. Ample laboratory research has demonstrated an attentional bias to affectively salient stimuli (e.g. emotional faces) (*affect-biased attention*)^38^. For example, when images of faces with different expressions are displayed on a computer screen, humans tend to direct their attention to emotional ones^39, 40^ or prefer to look at simultaneously presented happy compared to neutral faces, but avoid looking at angry faces^41^. Even when unaware, people react differently to emotional faces, and are likely to avoid gazing at angry faces and gaze more to fearful faces compared to neutral faces^42^. Expressions provide an important social cue, requiring appropriate reaction to another person’s feeling. It is therefore crucial for humans to promptly detect and correctly identify emotions of other people during social interactions. Although facial expressions can be identified in the peripheral visual field^43^, the recognition of emotional expressions is significantly better when faces are foveated than when they are processed in the peripheral visual field^44^. Therefore, it may be of advantage for people to directly look at others who show emotions to collect additional information about the expression. In contrast, in social situations, people may particularly avoid direct gaze and the related, potentially more challenging, interaction with an emotional person. However, little is known about how emotional expressions affect gaze behaviour in real life. To our best knowledge, Gallup and colleagues (2014) published the only study demonstrating the effect of different emotional expressions in gaze-following during a real life situation. They found that pedestrians walking in social groups were more likely to follow the gaze of a confederate displaying emotional facial expressions indicative of threat than of a confederate showing a neutral expression^45^. In summary, although emotion recognition is particularly relevant in live situations, research in this area is lacking. The current study will fill this gap by investigating the effects of another person’s emotional expression on gaze in a real-life social situation.

Individuals may differ in their gaze in social situations, based on personal characteristics and cultural differences. Incidental evidence suggests that people with poor social skills turn their head more towards a confederate than people with better social skills^26, 46^. The findings of the current study will therefore have implications for clinical groups. In particular, a deficit in social skills and Theory of Mind exists in people with *autistic traits*^47–50^. People with high autistic traits equally look at an experimenter when they believe them to be present in a live video chat as when they believe to be viewing a video recording, while people with low autistic traits would look less at the experimenter in the live condition^46^. However, if autistic people are engaged in an interaction, they look significantly less at the interaction partner than people with low autistic traits^46^. The atypical response to social stimuli is even one of the criteria defining autism according to the Diagnostic and Statistical Manual of Mental Disorders (American Psychiatric Association, 2013)^51^. Therefore, autistic traits will be measured in the current study. S*ocially anxious individuals* may particularly avoid gazing towards others in social situations. For example, they showed an attention bias away from emotional faces in a dot probe task, specifically when they were told their social skills would be judged^52^. This hypervigilance-avoidance to emotional faces is dependent on the context (e.g. social stressor, such as performance judgment) and time of exposure to the emotional stimuli^53, 54^. Most of the experiments on attention in social anxiety were performed in laboratory settings, so reactions may differ in realistic situations^54^. Consequently, individual differences in social anxiety level will be controlled too. Besides personal characteristics, humans are also influenced by customs and social rules of their cultural group. In other words, c*ultural differences* also affect social attention^55, 56^. Research comparing traits and behavior of people belonging to different societies have found that there are specific intragroup characteristics common to their members. For example, some populations tend to be more collectivistic (i.e. feel more in-duty to the group) and others more individualistic (i.e. value personal independence more)^57, 58^. These cultural values can impact the view of the self (self-construal). Individuals in individualistic societies will have a more independent view of the self, and people from collectivistic societies a more interdependent self-construal, i.e. viewing the self as more connected to others^59^. The perception of the self affects both people’s cognitive and affective processes^55, 56^, and their neural processing^60, 61^. In a recent study, Lo and colleagues (2021) investigated the influence of independent or interdependent self-construal primes on attention shifting in response to group gaze cues in a multi-gaze cueing task. Both European Canadians (more independent) and East Asian Canadians (both interdependent and independent) completed the task; however, only the latter were affected by the primes. These results suggest that social attention, which is the subject of the current study, can be influenced by cultural background^59, 61, 62^. Overall, considering the importance of cultural differences in attention research, the current study will additionally collect information about self-construal characteristics (independent vs. interdependent) using the Self-Construal Scale^63^. We will openly share our data, enabling future investigation of the effect of cultural differences on social attention in realistic settings by international researchers.

In summary, the current study aims to investigate the effect of emotional expressions of others on gaze patterns in live compared to laboratory situations. For this purpose, we will implement a similar waiting room paradigm to the original task by Laidlaw et al. (2011)^26^. Participants will either be waiting in a room with another person (confederate) or see a video of the confederate on a screen. Covert and overt attention will be measured with eye-tracking through indirect and direct looks towards the confederate, and any verbal social interaction during the presence of the confederate will be recorded. The novel addition to the original paradigm is that the confederate will display positive, negative and neutral emotional expressions during the experimental session. We will examine the interaction of emotional content, manipulated through the facial expression of the confederate, and social context, manipulated as the confederate being present in real life (social situation) or visible on a computer screen (non-social) (see Table 1). As there may be differences in gaze behavior between people with different levels of autistic traits and social anxiety, these traits will be measured and controlled for in the analyses. Based on previous research comparing gaze in social and non-social situations^26, 33^, we expect participants to look longer at the confederate in the video than in the social condition, corroborating the importance of the dual function of gaze in social situations. In other words, to avoid social interaction and respect social rules, participants will not look as much towards the physically present confederates than to their videos. Although emotional expressions also have been shown to affect gaze patterns in numerous studies^34, 35, 38^, the pattern to be expected is less clear. Overall, two aspects might influence participants’ gaze behaviour towards emotional expressions: their evolutionary relevance (especially of angry or fearful faces), and social context. According to emotion research in a laboratory context^39, 40^, participants should gaze more towards positive than towards neutral faces, as positive expressions are more relevant for behavioural decisions and motivational states^64, 65^. This outcome is in line with the assumption that positive expressions (e.g. happy faces) have less ambiguous communicative intent^66^ and increased perceptual saliency that makes them more noticeable than neutral or negative expressions^67^, regardless of the cognitive load of the situation^68^. The effect of negative expressions is more ambiguous, meaning that they may either attract more or less gaze than neutral expressions. From an evolutionary perspective, looking more at negative expressions compared to neutral ones, would be in line with findings that negative expressions (e.g. angry faces) rapidly capture attention^69^, and that delayed disengagement from them is important for gathering more information and threat processing^34, 35, 70^. In contrast, participants may avoid looking at negative stimuli either to regulate their own emotions, or in accordance with an automatic avoidant behaviour towards threatening stimuli^71–73^. The direction of emotion effects may furthermore vary depending on the social context. In the video condition, participants will not have the chance to interact with anyone, and consequently they do not have to consider social rules. Hence, participants may look more at emotional expressions^71, 72^. In contrast, in the social condition, participants must consider social rules; therefore it is either possible that they look less at emotional expressions to avoid interaction or that the social and evolutionary relevance becomes even more salient in social situations and that participants consequently look at emotional expressions even more. Our design is the first to allow comparing emotion effects based on social context. We will further control for individual differences, as the effects of social context may be smaller for people with high compared to low autistic traits, due to atypical gaze regardless of condition when no social interaction is expected^26, 46^. But we expect larger effects for people with high compared to low social anxiety, because of increased gaze avoidance in social situations^52, 74^. Lastly, we hypothesize that, in the social condition, interactions between the participant and the confederate have a higher probability of being initiated during displays of positive expression, since happy faces, for example, have a strong motivation power^75, 76^. Yet, individuals with high social anxiety and autistic traits should still avoid social interaction regardless, because of impaired social interactions^77–80^. In conclusion, this work will be the first to directly investigate differences in emotion-driven attention between live social situations and non-social laboratory situations, furthering the understanding of social attention to emotional faces in real life situations.

**Table 1:** Design table.

Question	Hypothesis	Sampling plan (e.g. power analysis)	Analysis plan	Interpretation given to different outcomes
Does social context influence gaze?	Participants will fixate the confederate longer, faster and more often in the non-social than in the social condition	Minimum of 19 participants needs to be tested per condition (Cohen’s *d* = 1.21, power = 0.95 and alpha error = 0.05)	If the dataset has a normal distribution, we will perform a parametric unpaired *T* test. Otherwise, we will use a Wilcoxon test	The difference between the sociality conditions will be considered significant when p < 0.05, in this case we will conclude that social context affects gaze. Otherwise, additional Bayes factors will be computed to compare the likelihood of the Null to the alternative hypothesis
How do different facial expressions affect overt attention towards the confederate?	Proportional looking times, number of fixations, and latency of the first fixation to the confederate will be longer, more frequent, and faster to positive than neutral expressions. Gaze patterns to negative expressions will differ from neutral ones depending on the evolutionary variables standing out: gathering more information or avoidance of threat	A minimum of 22 participants needs to be tested per condition (Cohen’s *f* = 0.605, power = 0.95, and alpha error = 0.05)	If model assumptions are met, a mixed ANOVA will be computed to investigate the effects of the facial expressions, sociality and their interaction on the mentioned dependent variables. Otherwise, we will perform a robust mixed ANOVA Post-hoc pairwise *t* tests will be computed to determine where the main differences lie	If the interaction is significant (*p* < 0.05), we will conclude that effects of expression differ depending on social context If the effect of facial expression is significant (*p* < 0.05), we will conclude that emotional expressions affect gaze patterns
How does expression interact with sociality?	In the social condition, expression effects will be stronger than in the non-social condition			
Does covert attention to the confederate differ depending on sociality and expression?	During the display of emotional expressions, the number of covert fixations may be higher and looking times longer to the confederate than during neutral expressions	A minimum of 22 participants needs to be tested per condition (Cohen’s *f* = 0.605, power = 0.95, and alpha error = 0.05)	If model assumptions are met, we will compute a mixed ANOVA to investigate the effects of facial expression, sociality and their interaction on the covert gaze variables. Otherwise, we will perform a robust mixed ANOVA	If the interaction is significant (*p* < 0.05), we will conclude that expression effects differ depending on social context. If the effect of sociality is significant (*p* < 0.05), with more covert gaze in social situations, we will conclude that attention is shifted covertly more often in social situations. If the effect of expression is significant (*p* < 0.05) we will conclude that expression affects covert attention
	Covert attention will occur more often during social than non-social conditions			
In the social condition, does participants’ initiation of a conversation depend on the confederate’s expression?	There is a higher probability of interaction initiation during displays of positive expression	A minimum of 5 interactions per facial expression must occur	If the minimum of 5 interactions per facial expression occurs, we will conduct a Chi-squared test to compare positive expressions with negative and neutral ones. Otherwise, we will share the observations qualitatively	If the initiation significantly differs between positive and other expressions (*p* < 0.05), we will conclude that positive emotional expressions facilitate interaction initiation
Are the findings independent of individual differences in autistic traits and/or social anxiety traits?	Effects of social context may be smaller for people with high compared to low autistic traits, but larger for people with high compared to low social anxiety. We hypothesize that a model controlling for these individual differences will fit the data better than a model excluding them	The sample sizes computed above will be used and the data added to an additional model controlling for individual differences	If the model assumptions for linear mixed models are met, we will compare a model (1) with facial expression, sociality and their interaction, with a model (2, 3) using facial expression, sociality and their interaction, and one of the questionnaire scores (either the Autism Quotient or Social Interaction Anxiety) as predictors . In addition, a model (4) with facial expression, sociality and their interaction, and both questionnaire scores will be compared to the models (2, 3) including only one of the questionnaires as predictors. These models will be computed for the outcome measures for overt and covert attention In case there are model violations, we will compute a robust linear mixed model	If the model with one or more individual differences factors fits the data significantly better than the model without individual differences (measured through significantly lower AIC, as determined through a model comparison using the *anova* function in R), we will conclude that individual differences in the factor contributing to a better model fit play a relevant role

## Methods

### Ethics information

The study was approved by the local ethics committee of Georg-August-University Goettingen (reference number 240) and affirmed by the ethics committee of Friedrich-Alexander University Erlangen-Nürnberg (confirmation number 361_20 B) and was conducted in line with the Declaration of Helsinki. Consent was obtained from participants before they participate in the study, after they were partially informed about the procedure (use of eye-tracking) but not about the presence of a confederate. Participants were asked to sign a second written consent after the full debriefing regarding the confederate/video of the confederate. They were reminded of the option to have their data deleted during debriefing. The confederate, and first author of the current study, has given informed consent for publication of identifying information/images (such as in Figure 3) in an online open-access publication.

### Design

In a 2 × 3 mixed design, the sociality condition (social or non-social) was controlled between participants. The expression of the confederate (positive, neutral, negative) was manipulated within participants. Scores of the Autism Quotient questionnaire^81^ and the Social Interaction Anxiety questionnaire^82^ were measured.

The effect of independent variables on (1) proportional gaze duration to the upper body and face of the confederate, (2) number of fixations to the upper body and face of the confederate, (3) latency of the first look to the confederate, (4) covert orienting to the confederate, and (5) possible initiation of verbal interactions were measured. As data accuracy was sufficient, we also explored the proportional fixations to the clipboard the confederate was holding to “fill out questionnaires”.

### Sampling plan

#### Sample size calculations

The sample size was determined through power simulation analysis in RStudio Version 1.4.1103 (RStudio, Boston, United States). The R script is included in the supplementary material (Supplementary Methods “Sample Size Calculation”). First, we performed a power analysis for the between participants effect of the sociality condition (Social vs Non-social) using the *pwr.t.test* function from the ‘pwr v1.3–0’ package^83^. As our hypotheses are based on the study by Laidlaw et al. (2011)^26^, we extracted their effect sizes (Cohen’s *d*), which were 1.21 for effects on looking time and 1.32 for number of fixations. With an alpha error of 0.05 and a power of 0.95, 19 and 16 participants need to be tested per condition, for each measure respectively.

As the current study was the first to investigate differences in looking time to emotional faces in live social situations, the effect sizes for the expected expression effects are less clear. We addressed this issue by modelling the required sample size in two different ways.

Firstly, we modelled required sample sizes for a mixed analysis of variance (ANOVA) based on the effects observed in the study by Laidlaw et al. (2011). A test using the *wp.rmanova() function* from the ‘WebPower v0.5.2’ package^84^ was conducted, for a 2 × 3 mixed design including the between-subject condition Sociality (Social vs. Non-Social), and within-subject condition Expression (Positive vs Neutral vs Negative). With a power of 0.95, Cohen’s *f* = 0.605 (computed from the effect size of Laidlaw et al. (2011))^85^, and alpha error = 0.05, 38 participants in total are required to observe the between-subject effect of social condition and 44 individuals *in total* are required to detect an effect of the within-subject factor emotional expression and of the interaction between emotional expression and sociality. To allow for full counterbalancing of the order of three displayed expressions, 24 participants need to be tested per group (48 overall).

Secondly, we simulated potential datasets based on the study by Laidlaw et al. (2011) comparing live and non-live situations and the study by Gamble et al. (2010) investigating emotion effects^26, 41^. To create a model dataset, we extracted the information about the mean and SD of looking time from the live (i.e. social M = 0.83s) and videotaped (i.e. non-social, M = 14.9s) conditions from Figure 2 of the Laidlaw et al. (2011) study using WebPlotDigitizer v. 4.4 software (Pacifica, United States)^86^. These values were used as a baseline for the main difference between conditions. As SDs differed between conditions, we used the SD of the videotaped group (SD = 1.9), but planned to use non-parametric tests if the homogeneity of variance assumption should be violated in our sample.

To our best knowledge, looking times to emotional expressions during live interactions have not directly been measured. Consequently, we based our expected differences in looking time between emotional expressions on a laboratory study by Gamble et al. (2010)^41^, which measured proportional looking times to simultaneously presented emotional (happy or angry) compared to neutral faces in a free-viewing paradigm. We extracted the proportions from their Fig. 1, using the WebPlotDigitizer v. 4.4 software. Healthy participants looked at happy faces 55% of the time, compared to the total (happy + neutral faces), whereas they looked at angry faces 47% of the time. Based on the computed mean and SD values, 1000 data sets were simulated, with different sample sizes (from 10 to 150 in steps of 10), SD = 1.9, and an alpha error of 0.05. Using the modelled datasets, a mixed ANOVA (*anova_test()* from ‘rstatix v0.7.0’ package)^87^, was conducted to investigate effects of sociality (Social vs. Non-Social), expressions (Positive vs. Neutral vs. Negative) and their interaction. We plotted *F* values, *p* values and effect sizes as a function of sample size. From the partial eta-squared provided by the R function, we additionally calculated Cohen’s *f* for better comparison between the two tests^85^. The simulations show, primarily, that even with the smallest sample size of n = 10 (per sociality condition) the expected effects would be significant with p considerably lower than 0.05. In addition, we investigated the effect sizes to be expected based on the simulated data. According to Cohen (1988)^85^, an *f* of 0.10 is considered a small effect, 0.25 a medium, and 0.40 a large effect. Considering 48 participants in total, 24 for each condition, and SD = 1.9, we expect a very large effect of condition (Cohen’s* f* = 6.7, *p* = 1.4 × 10^−34^), and large effect sizes for emotion (Cohen’s *f* = 0.74, *p* = 3.2 × 10^−6^) and the interaction between emotion and condition (Cohen’s *f* = 0.66*, p* = 2.6 × 10^−5^).

**Figure 1:**
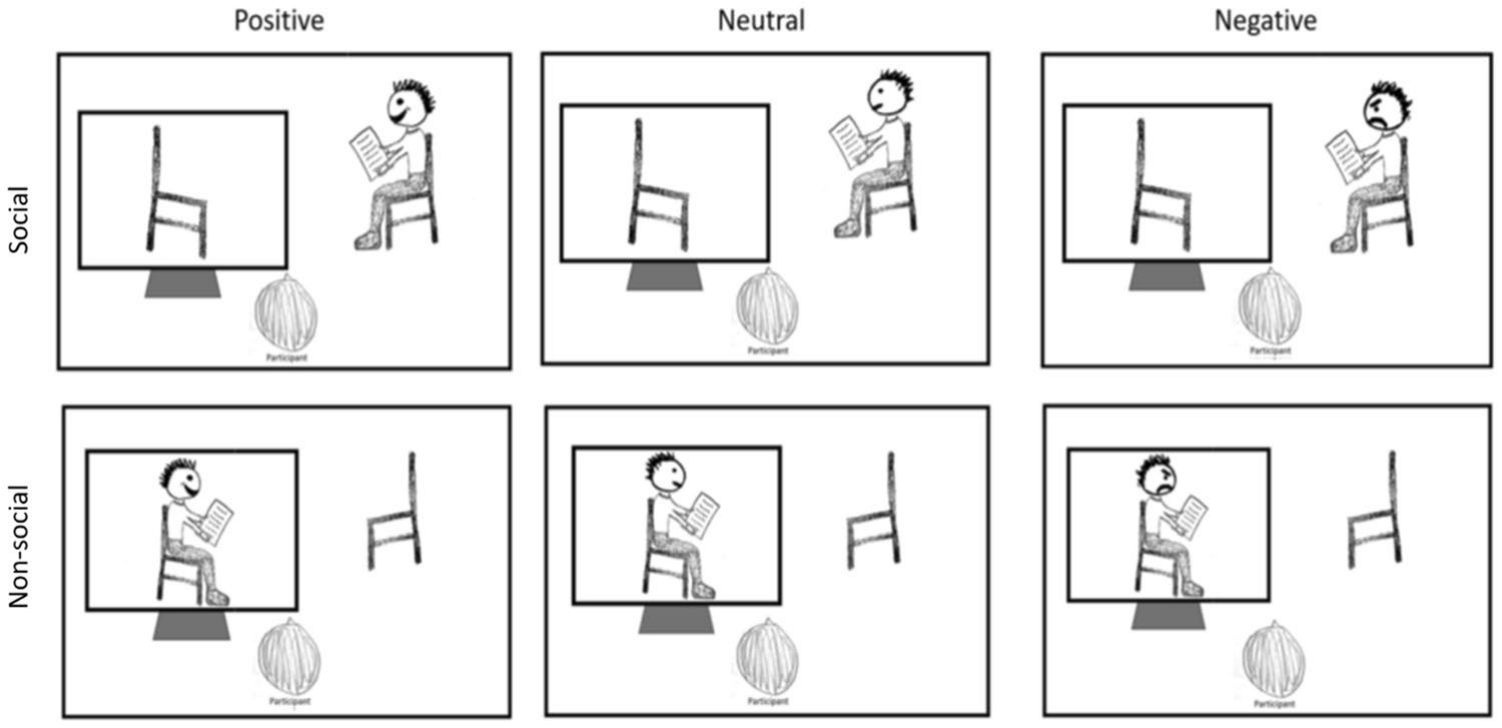
Experimental design. There were two sociality conditions: (1—Social) The participant was in the presence of the confederate, who was sitting with a clipboard, pretending to fill out a questionnaire. In this case, the reference object was the computer screen. (2—Non-social) The participant saw the video of the same confederate filling out the questionnaires, which was recorded during a previous social session. In this condition, the chair was the reference object. In both conditions, the confederate displayed a positive (i.e., smiling), negative (i.e. frowning/annoyed) or neutral facial expression, as if it was a reaction to the content of the questionnaires. The order of expressions was counterbalanced between participants.

In conclusion, based on all the power analyses and simulations, with the most conservative criteria and considering the possibility of full counterbalancing, 48 participants will be sufficient to observe the expected effects (i.e. 24 in the social group, and 24 in the non-social group). If participants dropped out due to experimenter error (e.g. not following the rehearsed script during the experiment), due to technical challenges related to mobile eye-tracking, due to the participant initiating a conversation (i.e. verbal interaction) with the confederate during the test phase, or due to the participant guessing the aim of the experiment, additional participants were to be tested until we reached the predetermined number of participants. We implemented a stopping rule to test only up to three times the required number of participants per sociality condition (72 per condition), as such large drop outs would be unexpected based on the literature.

#### Participant characteristics

An age range between 18 and 35 years was chosen, as gaze patterns may change with older age^88^. Furthermore, to be included, participants should have no previous neurological and/or psychological disorders, have normal vision (i.e. no glasses, which cannot be combined with the mobile eye-tracker), and be native German.

#### Eye-tracking

Eye movements were recorded using a Pupil Invisible Eye-Tracker (Pupil Labs GmbH, Berlin, Germany). These mobile eye-tracking glasses simultaneously record gaze using two eye cameras with 200 Hz sampling rate, as well as a scene camera with 30 Hz sampling rate. The eye-tracker does not require separate calibration, because it implements an automatic calibration algorithm. However, at the end of the experimental session, to investigate the accuracy of the mobile eye-tracker, the participant were asked to look at a printed calibration marker (v0.4 marker design, Pupil Labs) in a 5-point calibration type disposition from approximately 2 m distance.

#### Procedure

Upon arrival, the participants were partially informed about the aims of the study. They were told that an eye-tracker would be used to monitor their eye movements and that they would be shown photographs and paintings; however, they were blind regarding the presence of a confederate (or video of a confederate) in the waiting room. They signed a consent form on a clipboard. Participants were asked to store their phone and personal belongings in a safe location for the duration of the study to ensure that they were not distracted by their phones. The experimenter set up the eye-tracking glasses, and put away the gaze recording phone attached to the equipment within a pouch around the participant’s waist. Subsequently, the experimenter stated that they needed to finish setting up the experiment in the other room and asked the participant to have a seat on a designated chair and wait in a waiting room.

Half of the participants were led to a room by the experimenter, in which a confederate (played by a researcher) was sitting with a clipboard such as the one the participant just received her or his documents on, pretending to be another waiting participant (social condition). Upon entrance of the participant, the confederate nonverbally acknowledged his/her presence by looking at his/her face and nodding (see the 'Behavioral Script' on Supplementary Methods). Subsequently, the confederate started to pretend to fill out the questionnaires again and did not gaze towards the participant again. The other half of the participants were led to the same room, in which a screen showed a muted video of the same confederate, recorded during a previous social session (non-social condition). Papers were lying in front of the monitor, to mimic a situation in which a researcher left “on” the video they were coding.

The confederate filled in a questionnaire on the clipboard and while doing so displayed either a positive (i.e. smiling), negative (i.e. frowning/annoyed) or neutral facial expression (Fig. 1). When the confederate turned the page of the questionnaire, the facial expression changed. To ensure approximately equal duration of each facial expression, the confederate read the same amount of text on each questionnaire page and practiced the time spent on it. Each facial expression was displayed once for approximately 1 min. This duration allows for sufficient time for emotional expression recognition (e.g. 50 ms is the minimum time required)^89^, while our pilot research (with 6 lab members blind to the aims of the study) also suggests that emotional expressions are still perceived as natural during this time window. In total, expressions were displayed once, totalling 3 min in the waiting room. Note that the exact timing could vary due to the natural situation. On average, each live condition was displayed for 68.06 s (minimum = 57, maximum = 101). The order of facial expressions was counterbalanced between participants. The confederate knew which emotion to display, as it was stated in the first sentence written on each questionnaire page. This furthermore allowed for a natural evolvement of the expressions, as it appeared to be in response to something the confederate read on the questionnaire. To create a natural appearance, the confederate looked up to the front three times according to a prompt written on their clipboard (one time during each type of facial expression). The confederate was filmed during each real-world session, with a Canon PowerShot SX740 HS camera (Canon Inc., Tokyo, Japan) located on a shelf on the wall approximately 85 cm above and 40 cm to the right of where the participant sat. In every video session, the video from one of the real world sessions was played to the participants. The first participants started the experiment with the social condition, in order to generate the video recordings of the confederate for the non-social condition. The confederate wore the same outfit (plain jeans and a black shirt) in each session to avoid visual confounds.

Gaze towards the face and upper body of the confederate compared to gaze to the surrounding area were measured. The location where the confederate appeared in each condition served as the reference object for the opposite condition: the chair for non-social group, and the screen for the social group. If the participant verbally initiated a conversation (i.e., started talking to the confederate), the confederate naturally replied to the participant. Any attempts of the participant to interact verbally with the confederate during the test session were registered and trials in which conversations occur were additionally analysed separately, in the analysis of “initiation of interaction”. These trials were excluded from the overall gaze analysis, as the interaction would lead to deviations from the confederate’s scripted actions, when they respond to the participant. Note that the confederate did not directly look at the participant during the waiting room situation so that a non-verbal initiation of conversation would not be possible.

**Figure 2:**
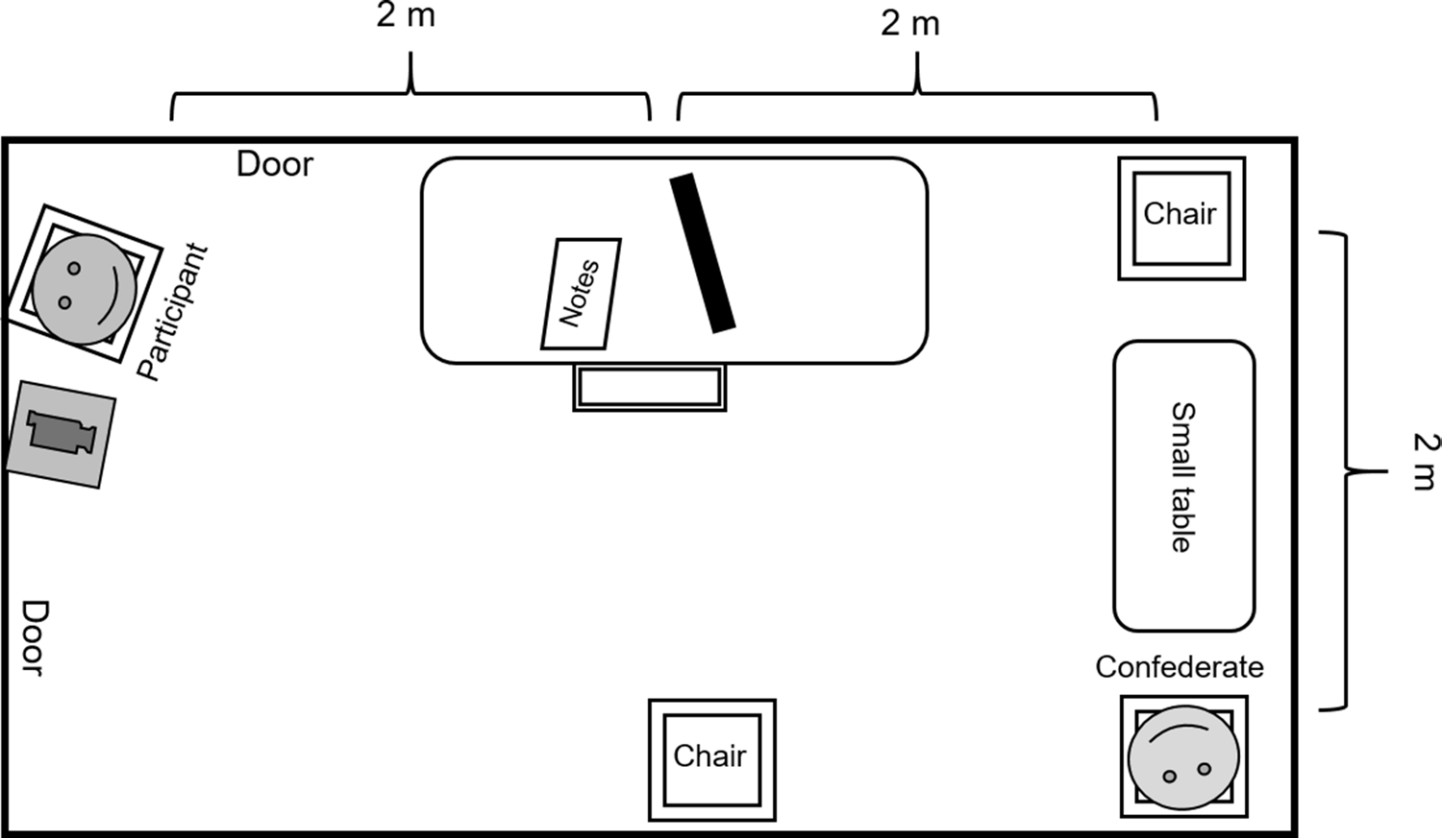
Disposition of the waiting room. The participant sat at the left upper corner of the room. In front of the participant, there was the desk, with a chair, the monitor (approximately 2 m from the participant) and papers in front of it. In the non-social condition, the monitor was turned on, displaying a video of the confederate (upper body and head only). In the social condition, the confederate sat in the right corner, approximately 4 m horizontally and 2 m vertically from the participant. The camera recording the confederate was situated on a shelf, diagonally above the participant (about 40 cm horizontally and 80 cm vertically).

Eye-movements may differ between sociality conditions if the confederate looks smaller on the screen than in reality (in that case the real-life confederate might be explorable through peripheral vision, while the image on the screen would require foveal fixation). To account for this, the retinal size of the confederate was kept approximately constant between the real-life and the video condition by (1) placing the monitor closer to the participant, than the confederate, and (2) the recordings in the video condition only showing the confederate’s upper body filling the whole screen. Note that the natural variation in position of the confederate induced some variation in the retinal size, despite our efforts to keep it constant. In the live condition, the visual angle was on average 9° 12′ 0.54'' and in the video condition 9° 51′ 0.55'', with some variations due to the placement of the confederate and camera (max = 10° 27′ 0.36' and min = 9° 15′ 0.87''). The confederate sat diagonally to the participant (around 4m vertically and 2 m horizontally), and a 27’’ Philips 278B1 monitor was positioned on the table in front of the participant (approximately 2 m away). The chair of participants was positioned so that they were facing in between the monitor and the confederate (see Fig. 2 for the layout of the room).

Finally, the experimenter returned to collect and instruct the participant to freely view different photos and paintings hanging in the adjacent room. The experimenter told the participant that he or she will wait in the other room for the participant to finish the task. After completing the decoy task, the participant was led back to the waiting room to fill in the Autism Quotient questionnaire^81^ and the social interaction anxiety questionnaire on a clipboard^82^. Participants also filled out the Self-Construal Scale (SCS)^63, 90^, which assesses independence and interdependence traits, which may be relevant for the comparison of the current native German sample with future cross-cultural studies on social attention. Finally, the experimenter orally asked questions from a debriefing questionnaire to investigate the participant’s awareness of the aims of the study. The questionnaire included the following questions: (1) what the participant believes the aim of the study was, and (2) whether they noticed anything unusual during the study. In the video condition, we further asked (3) whether they noticed anything unusual about the video playing in the monitor during the study and (4) whether they noticed that the video playing on the monitor was part of the experiment. During the social condition, we additionally asked (3) whether they noticed anything unusual about the people who were present during the study and (4) whether they notice that the other person in the room was part of the experiment. Participants were asked to sign a second written consent after the full debriefing regarding the confederate/video of the confederate. They were reminded of the option to have their data deleted during debriefing.

### Analysis plan

#### Pre-processing

Participants’ gaze was coded from the eye-tracking recordings by one of the authors and independently by a research assistant to determine inter-rater reliability. For this purpose, the recordings, with the determined gaze position overlayed, were replayed frame by frame. The first frame after the confederate flips the page was considered the start of a specific condition, which ends with the last frame before the next turn of page. For each frame, it was coded (1) whether gaze was available or whether a blink or loss of gaze position occurred and (2) if gaze data was available, whether the participant looked at the face/upper body of the confederate, the clipboard that the confederate was looking at, the reference object, or another location in the waiting room. We obtained 99.3% agreement and Cohen’s kappa of 0.944 for the live condition, and 95.7% agreement with Cohen’s kappa of 0.892 for the video condition. As the inter-rater reliability was consistent, we used the coding from the most experienced experimenter as the basis for the rest of the pre-processing and statistical analyses.

Within each sociality condition, the coders determined the latency of the first look to the confederate by subtracting the time stamp of the start frame from the time stamp of the frame in which the first look at the confederate occurred. Coders counted the number of direct looks, i.e. overt looks to the face or upper body of the confederate. Coders determined the duration of looks to each of the areas of interest by counting the number of frames that participants spent looking at each of them. They then calculated the proportion of looking time (i.e. number of frames) to the face and upper body divided by the overall looking time (i.e. number of frames with gaze to the face, the upper body, the clipboard, the reference object and other locations) for each expression condition.

Additionally, based on Dosso et al.’s study (2020), covert attention shifts were determined as instances when the participants turn their gaze towards, but do not fixate on the confederate^30^. In this case, the participant might have used their peripheral vision to observe the confederate, without a direct look (indirect look). It should be noted that this measure cannot guarantee that the participant was indeed covertly attending to the confederate rather than overtly attending to the current object of fixation and results should therefore be interpreted with caution. Comparably to overt attention, the number of covert fixations and their duration were coded for covert attention.

It was determined whether participants initiated a conversation or not and if they did so, in which expression condition the initiation occurred. If the participant started talking to the confederate, the data from this participant were not used in the main analysis, but only included in the specific analysis about “initiation of interaction”.

When the data quality allowed to distinguish looks to the confederate and the clipboard, as an exploratory analysis, we measured gaze-following to the clipboard^45^. Instances when the participant looked at the face of the confederate and subsequently looks at the clipboard the confederate was holding were considered “gaze-following”. The number of gaze-following events were counted for each condition. This way, we expected to measure natural gaze-following of the participant toward to the confederate. The number of looks to the clipboard, right after fixating the face of the confederate divided by the number of frames of each expression were computed. In addition, we computed the total number of fixations to the clipboard, to also include possible gaze-following after covert attention to the confederate.

### Analysis

#### Demographics

In total, 57 participants were tested until we reached 48 participants with viable data (40 female, 7 male and 1 diverse, mean age ± SD: 20.5 ± 2.99) (see Supplementary Methods “Analysis Script”). Three participants were excluded because of technical error with the mobile eye-tracking, two participants interacted verbally with the confederate, and four participants due to experimenter’s error: 1 was shown the wrong video, and 3 watched one of the displayed expressions for less than one minute. In addition, three participants used their phones at some point during waiting room in the live condition, despite being told to leave their phones with their belongings outside. They were not excluded, because they did it regardless the instructions of the experimenter; hence, the originally defined exclusion criteria were not violated. We did not foresee this participant behaviour; in the camera recordings it can be observed that one participant placed his phone on the table when instructed to do so by the experimenter but snatched his phone back behind her back when he followed her to the waiting room. The three participants checked their phone in total for 11, 50 and 195 s. Additional exploratory analyses excluding the participant looking the longest to their phone had equal results to the ones including the said participant (see Supplementary Methods “Analysis Script”).

#### Model assumptions

Violations of model assumptions were investigated for all gaze data and latency data. First, outliers were detected using the function *identify_outliers* from the ‘rstatix’ package^87^.

For the mixed ANOVA, we checked normality separately for each group on the “raw” data (using *shapiro_test* from ‘rstatix’)^87^, and subsequently, we plotted the QQ plot for the correlation between our data and the normal distribution (*ggqqplot()* from ‘ggpubr’)^91^. Additionally, we tested whether the variance of residuals was homogenous for all test conditions (using *levene_test* from ‘rstatix’)^87^. As the data violated most of the assumptions, corresponding non-parametric tests were conducted.

For linear mixed models, we investigated whether model residuals were normally distributed (using *check_normality* from ‘performance’)^92^ and the variance of residuals was homogenous for all test conditions (using *check_homogeneity* from ‘performance’)^92^. Tests for collinearity were performed using the function *check_collinearity* from the package ‘performance’^92^. Violations of homoscedasticity were checked with the function *check_heteroscedasticity* (‘performance’)^92^. Finally, we planned to use the function *check_model()* to get an overview of the model via plots (‘performance’)^92^. However, as this function was functional when we registered this report but no longer functional when we finished data analyses, due to updates, this overview was done manually instead.

In case there is a serious violation of those parameters, corresponding non-parametric tests were planned to be conducted, which was the case for our data.

#### Gaze to the confederate

First, we tested whether social context influences gaze to the confederate (first look latency, gaze proportion to the confederate (face and upper body), and number of looks to the confederate) by using a parametric unpaired *T* test (*t.test* function from ‘stats’)^93^. To calculate the effect size, the *cohensD* function from package ‘lsr’ was used^94^. As the data was non-parametric, we computed a Wilcoxon test (*wilcox.test* from ‘stats’) and calculated its effect size with the *wilcox_effsize* function from ‘rstatix’^87, 93^.

Mixed ANOVAs were computed using the *anova_test* function (package ‘rstatix’)^87^ to investigate the effect of expression, sociality condition and their interaction on the following dependent variables: first look latency, gaze proportion to the confederate, and number of looks to the confederate. Post-hoc pairwise t-tests were conducted to follow up observed effects using the functions *adjust_pvalue* and *pairwise_t_test* from ‘rstatix’^87^. We observed that the adjust_pvalue was not needed, because we could specify in the pairwise_t_test the *p* value to be adjuted to Bonferroni calculation. As the model assumptions were violated, we performed a robust mixed ANOVA using trimmed means (*bwtrim* from the ‘WRS2’ package)^95^. Because the *bwtrim* function does not offer effect size, to estimate it we extracted the general eta squared from the *anova_test* function whenever possible.

#### Covert attention to the confederate

To determine differences in covert attention as a function of social and non-social condition and facial expression displays, we computed two mixed ANOVAs. The number of covert fixations (ANOVA 1) and their duration (ANOVA 2) were included as dependent variable and sociality, facial expressions, and their interaction as independent variables in the model. R functions and procedures were carried out as described above.

#### Effect of individual traits in gaze patterns to the confederate

To control for individual variability in autistic and social anxiety traits, an additional linear mixed model was computed for each outcome measure, using the *lmer* function of package ‘lme4′^96^. In these models, gaze measures were the dependent variable. Sociality, facial expressions, and their interaction as well as results for autistic traits and social anxiety traits were included as predictors (i.e. fixed effects) and random intercepts were included for subjects. The model including both personality traits was compared to models including only one of the traits, as well as a model excluding all personality effects, to determine whether considering these effects leads to a better model fit. If it is indeed a better fit, the effects described above were compared to the effects of the model including the relevant personality traits. To run these model comparisons, we used the *anova()* function from the ‘stats’ package^93^. As there were violations of the model assumptions, we fitted the models as robust linear mixed models by using the function *rlmer* (‘robustlmm’)^97^. To compare the robust models, the function *compare_performance* the same way described above (package ‘performance’)^92^.

#### Initiation of interaction

We noted every verbal interaction initiated by the participant with the confederate during the social condition and computed which expression preceded the interaction. In case at least five interactions occur after each facial expression^98^, we would run a Chi-squared test (using the *chisq.test* function from the ‘stats’ package^93^) in order to test whether there is a difference among frequencies of interaction depending on the current facial expression of the confederate. As less than five interactions occurred for each facial expression, we reported them qualitatively in the results section.

#### Exploratory analysis: measure of gaze-following-proportion of looks to the clipboard

We originally planned that when data quality allowed to distinguish looks to the confederate and the clipboard, a mixed ANOVA would be used to investigate whether the proportion of gaze-following or total looks to the clipboard differ among valence of facial expressions and sociality conditions and any effects would be followed up with post-hoc *t* tests. Although data quality allowed this observation, there was not enough data of gaze-following for a formal analysis (13 incidents occurred for only 9 participants); therefore, we reported them qualitatively in the results section.

## Results

As our data violated most assumptions for parametric tests, we described the results on the non-parametric tests, unless we state otherwise. Both parametric and non-parametric had similar results that can be checked at the Supplementary Methods “Analysis Script”.

### Gaze to the confederate

#### First saccade latency to the confederate

We found no significant difference in the first saccade latency during social and non-social condition, *W* = 304.5, *p* = 0.549, *r* = 0.088. Furthermore, we confirmed that there was no effect of sociality, *F*(1, 10.44) = 0.47, *p* = 0.508, expression, *F*(2, 7.37) = 2.80, *p* = 0.124 , nor their interaction, *F*(2, 10.44) = 2.09, *p* = 0.191. Post-hoc analyses confirmed no significant differences between expressions per sociality condition.

#### Proportional looking time to the confederate

There was a large effect of sociality on the proportional looking time to the confederate, *W* = 1075, *p* < 0.001, *r* = 0.5071. In other words, participants looked longer to the confederate during the video condition (M = 0.119, SD = 0.152, 95% CI = 0.035) than during the live condition (M = 0.016, SD = 0.021, 95% CI = 0.005). In the mixed ANOVA, we confirmed the effect of sociality, *F*(1,15.79) = 12.56, *p* = 0.0027, η^2^G = 0.184, but there was no effect of expression, *F*(2,14.87) = 0.72, *p* = 0.504, η^2^G = 0.001, and no interaction, *F*(2, 14.87) = 0.26, *p* = 0.772, η^2^G < 0.001. Post-hoc analyses confirmed no significant differences between expressions per sociality condition.

#### Number of direct looks to the confederate

Participants also looked significantly more often to the confederate during the video condition (M = 16.261, SD = 16.666, 95% CI = 4.129) than during the live condition (M = 5.040, SD = 4.672, 95% CI = 1.327), *W* = 722.5, *p* < 0.001, r = 0.4761. Again, this effect of sociality was confirmed in the mixed ANOVA, *F*(1, 29.46) = 11.32, *p* = 0.0021, η^2^G = 0.127. However, no effect of expression, *F*(2, 20.59) = 0.05, *p* = 0.951, η^2^G < 0.001, or interaction was significant, *F*(2, 20.36) = 0.48, *p* = 0.623, η^2^G = 0.008. Post-hoc tests confirmed no significant differences between expressions per sociality condition.

### Covert attention to the confederate

**Figure 3:**
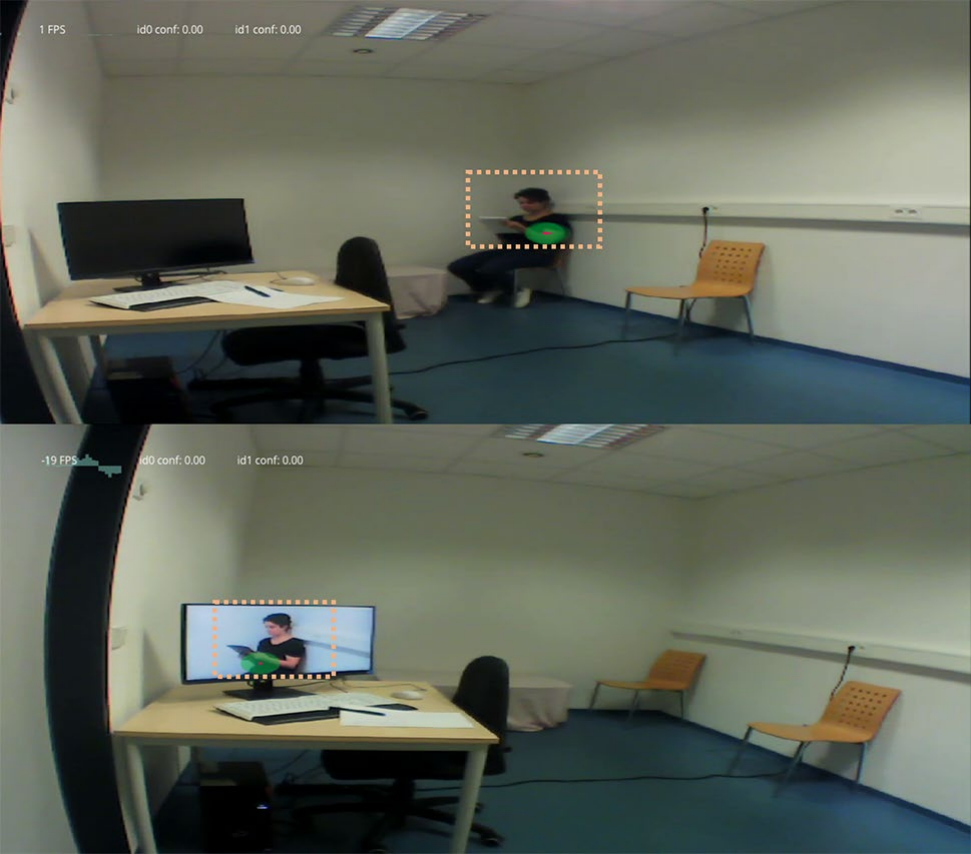
Example of live and video conditions. In the live condition (upper panel), the confederate sat diagonally to the participant, filling out the questionnaires. In the video condition (lower panel), a video of the confederate was streamed on the monitor screen. The dotted light orange around the confederate indicates the area around the body that was coded as “covert attention”. The red dot with green surrounding is the signal of the eye-tracker and we used the red dot to code the location of participants’ gaze.

As previously described, covert attention was defined as instances when participants looked in the direction, but not directly to the confederate (see Fig. 3, for the areas we considered as possible covert look locations). Participants covertly looked at the confederate significantly more often during the video (M = 16.109, SD = 17.606, 95% CI = 4.398) than the live condition (M = 6.072, SD = 5.702, 95% CI = 1.541), *F*(1, 28.17) = 11.54, *p* = 0.002, η^2^G = 0.113. The effect was independent of expression, *F*(2, 21.26) = 0.52, *p* = 0.599, η^2^G = 0.004 and there was no interaction, *F*(2, 21.66) = 0.05, *p* = 0.952, η^2^G = 0.005. Post-hoc analyses confirmed there were no significant differences between expressions per sociality condition. Participants also covertly looked at the confederate longer in the video condition, measured via the total number of frames (M = 194.84, SD = 240.59, 95% CI = 60.087) than in the live condition (M = 71.890, SD = 70.745, 95% CI = 19.125), *F*(1, 19.33) = 7.23, *p* = 0.014, η^2^G = 0.101. Again there were no significant effects of expression, *F*(2, 21.69) = 0.69, *p* = 0.511, η^2^G = 0.024 , or their interaction, *F*(2, 20.75) = 0.05, *p* = 0.947, η^2^G = 0.012 , which was confirmed by post-hoc analyses. For a comparison of covert and overt attention data measured in frames and proportional time, see Table 2.

**Table 2:** Mean proportion of looks and duration of looks (total frames) during overt and covert attention. This table shows the mean ± 95% confidence interval of the proportion of looks and duration of looks during overt and covert attention in each sociality condition and emotion expression displayed by the confederate.

	Live			Video		
	Positive	Neutral	Negative	Positive	Neutral	Negative
Proportion of looks – Overt attention	0.021 ± 0.011	0.014 ± 0.007	0.013 ± 0.007	0.122 ± 0.060	0.112 ± 0.067	0.124 ± 0.068
Proportion of looks – Covert attention	0.036 ± 0.013	0.026 ± 0.015	0.030 ± 0.020	0.132 ± 0.076	0.087 ± 0.045	0.083 ± 0.045
Duration of looks – Overt attention	82.10 ± 25.46	61.38 ± 34.23	71.61 ± 44.39	260.90 ± 146.64	160.72 ± 83.29	164.52 ± 84.73
Duration of looks – Covert attention	451.78 ± 142.96	474.34 ± 154.51	457.06 ± 147.08	399.68 ± 108.17	355.85 ± 101.86	375.81 ± 104.34

### Effect of individual traits in gaze patterns to the confederate

To investigate the effects of individual traits – autistic and social anxiety traits—we performed robust linear mixed models. Here we describe the results found in the best model fit, but all the models can be seen in the Supplementary Methods “Analysis Script”. Note that every model included participants as random effect. First saccade latency to the confederate was not included in the models, although it was previously intended, because the nature of this data turns the random intercept including participants irrelevant, as there is only one observation for each participant.

When considering *proportion of looking time to the confederate* as the dependent variable, the complete model including the interaction between sociality and expression, the SIAS scores and AQ scores as fixed effects was the best fit. The conditional R^2^ (R^2^ cond = 0.759) was higher than in the basis model (including only the interaction sociality x expression; R^2^ cond = 0.753), the model including only the basis and AQ scores (R^2^ cond = 0.754) and the model including the basis and SIAS scores (R^2^ cond = 0.753). As expected, we found significant effects of sociality condition (β = 0.07, *SE* = 0.01, *t* = 4.40, *p* < 0.001), but none of expressions (Neutral *p* = 0.891, Positive *p* = 0.341) nor their interactions (Video vs Neutral p = 0.317, Video vs Positive p = 0.223). In addition, there were no significant effects of AQ scores (β =  − 0.0007, *SE* = 0.001, *t* =  − 0.62, *p* = 0.538), and SIAS scores (β =  − 0.0001, *SE* = 0.0007, *t* =  − 0.18, *p* = 0.854). The more complex models also showed no significant interactions of questionnaire scores with any of the manipulated variables.

**Figure 4:**
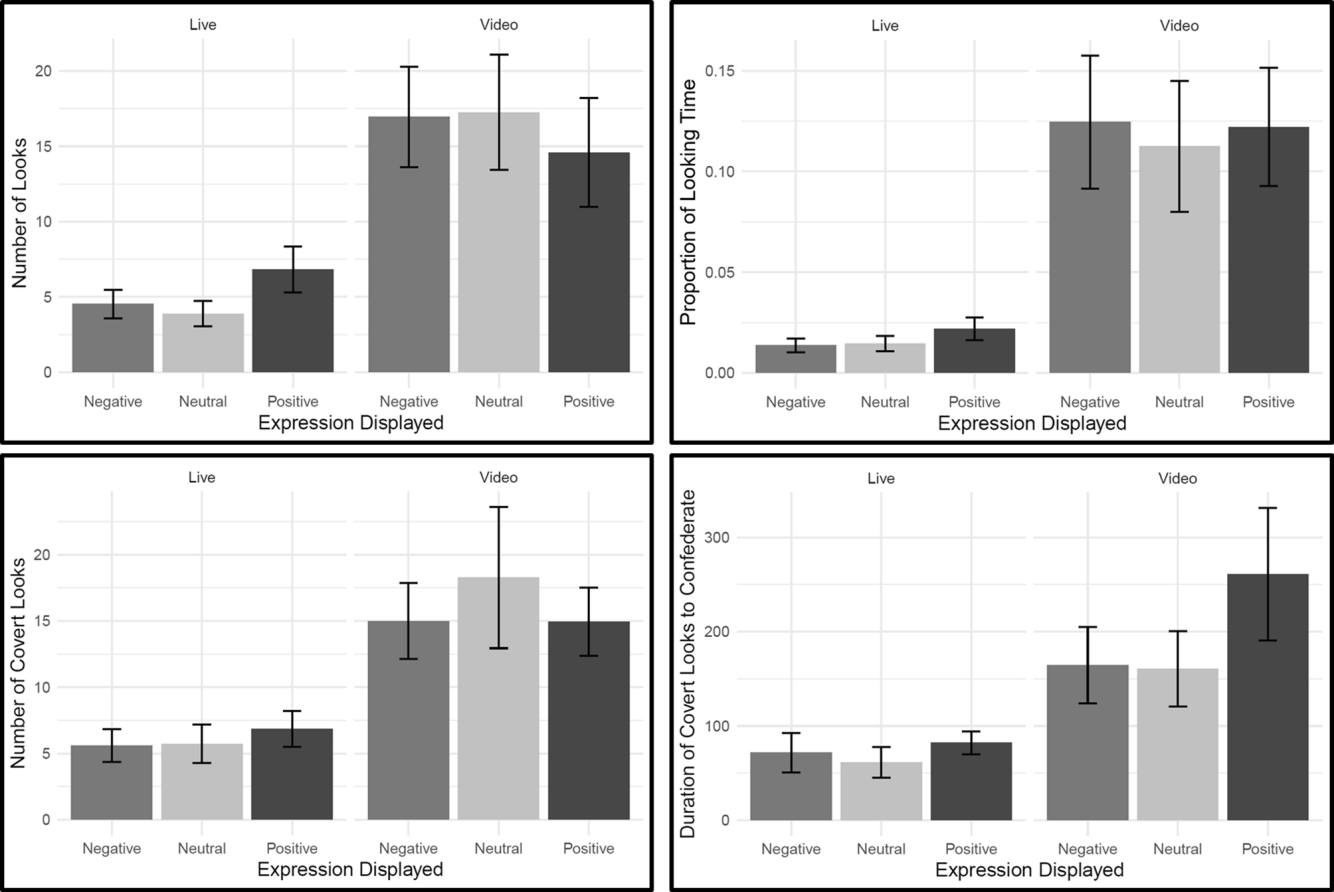
Visualization of gaze data to the confederate. The upper panel displays the number of direct looks to the confederate (left), and the proportion of looking time to the confederate (right). The bottom panel displays the number of covert looks to the confederate (left) and the duration of the covert looks to the confederate (right, y-axis represent the total number of frames). All the graphs include the bars per expression and sociality condition. The error bars represent ± standard error.

The *number of looks to the confederate* as dependent variable yielded a different best model fit. The model including the basis fixed effects plus the SIAS scores fit the best (R^2^ cond = 0.702) though similarly well as the simplest model with the basis fixed effects (R^2^ cond = 0.701), and the complete model including the basis effects plus the SIAS and AQ scores (R^2^ cond = 0.698). Again, the sociality condition had a significant effect, β = 7.66, *SE* = 2.13, *t* = 3.60, *p* < 0.001. Furthermore, the interaction of condition and positive expression was significant, β =  − 4.38, *SE* = 1.99, *t* =  − 2.19, *p* = 0.028. This result indicates that participants looked *more often* at the positive facial expressions compared to the negative facial expressions during the live condition. However, the opposite was the case in the video condition, in which participants looked *less often* to positive facial expressions compared to negative expressions (see Fig. 4). As this effect was unexpected, because we found no sociality and emotion interaction during the preregistered mixed ANOVA, we confirmed that the effect also occurred in the basis model, β =  − 4.33, *SE* = 1.99, *t* =  − 2.17, *p* = 0.030. There were neither effects of expressions (Neutral *p* = 0.472, Positive *p* = 0.193) nor the other interaction (Video vs Neutral *p* = 0.838). Again, we observed no effects of the SIAS scores (β =  − 0.04, *SE* = 0.08, *t* =  − 0.55, *p* = 0.581).

When including the *duration of covert looks* as the predicted variable, the most complete model (R^2^ cond = 0.481) fit the best. It was better than the basis model (R^2^ cond = 0.439), than the model including basis and SIAS scores (R^2^ cond = 0.455) and the model including the basis and AQ scores (R^2^ cond = 0.477). In this model, there was a non-significant tendency for sociality condition to predict the duration of covert looks (β = 48.67, *SE* = 28.10, *t* = 1.73, *p* = 0.083). As expected, there was neither an effect of expression (Neutral *p* = 0.845, Positive *p* = 0.396) nor their interactions (Video vs Neutral *p* = 0.940, Video vs Positive *p* = 0.526). Furthermore, no effects of AQ scores (β =  − 3.21, *SE* = 2.12, *t* =  − 1.51, *p* = 0.130) or SIAS scores were found (β = 1.01, *SE* = 1.24, *t* = 0.81, *p* = 0.415).

Finally, we computed the *number of covert looks* as the predicted variable, and compared the models. The best fitting model was the model including the basis fixed effects plus AQ scores (R^2^ cond = 0.502) which was better than the full model (R^2^ cond = 0.484), and the basis model (R^2^ cond = 0.476). We found that the effect of condition was significant, indicating that people indeed covertly looked more often to the confederate during the video condition (β = 7.18, *SE* = 2.29, *t* = 3.14, *p* = 0.002). However, there was neither an effect of expression (Neutral *p* = 0.994, Positive *p* = 0.452) nor any interaction (Video vs. Neutral *p* = 0.985, Video vs. Positive *p* = 0.306). Additionally we found no predicting effect of AQ scores (β =  − 0.12, *SE* = 0.14, *t* =  − 0.88, *p* = 0.376).

### Initiation of interaction

Only two participants interacted with the confederate during the live condition. One was a male participant, 20 years old, who interacted twice during the experiment. The first time he started conversion when the confederate was displaying a positive expression, asking what the confederate was smiling about. The second time occurred during the neutral expression, commenting about the amount of questions the confederate was answering in the questionnaire. The other was a 19-year-old female participant, who interacted with the confederate during the first expression (neutral) and asked if the confederate was also taking part in the experiment and mentioned the eye-tracking glasses. Both participants had low scores on the SIAS (30 and 20, respectively) and in the AQ questionnaire (16 and 20, respectively).

### Gaze-following to the clipboard

Because we did not have sufficient data for statistical analyses of gaze following, we offer qualitative observations about it. We had few instances of gaze-following, as shown in Table 3. The majority occurred during the video condition (7 participants compared to 2 in the live condition), and mostly during the neutral expression (5 times). However, as seen from the proportion of gaze following, participants spent more time looking at other regions of interest. On the other hand, participants did look at the clipboard in general, especially during the video condition (13 participants in the live condition and 19 in the video condition). The table showing the number of looks to the clipboard can be found in the Supplementary Methods “Analysis Script”.

**Table 3:** Gaze following to the clipboard. This table lists the number of times gaze-following occurred (“Number of Looks”) at the each expression, per participant (note that the majority of participants did not show gaze-following behaviour and are therefore not listed in this table). In addition, the proportion of gaze-following, calculated by dividing the number of times the gaze-following occurred by the total number of frames during the expression presentation (“Proportion of Gaze”).

Participant ID	Emotion displayed	Number of Gaze following	Condition	Proportion of Gaze following
LP11	Negative	1	Live	0.00058
LP19	Neutral	1	Live	0.00055
VP02	Positive	2	Video	0.00119
VP03	Neutral	1	Video	0.00057
VP04	Negative	1	Video	0.00057
VP05	Neutral	1	Video	0.00056
VP10	Neutral	1	Video	0.00060
VP10	Positive	3	Video	0.00190
VP11	Negative	1	Video	0.00056
VP20	Neutral	1	Video	0.00059

## Discussion

The aim of the current study was to investigate the influence of social context and emotional expression on attention in a naturalistic setting (waiting room). We found a large effect of social context –participants looked significantly longer and more often at the confederate in a video on a screen than when she was present in person – but no reliable effect of emotional expressions the confederate displayed (positive, negative or neutral) in the preregistered analyses. Furthermore, in contrast to our hypothesis, participants’ covert attention to the confederate was also higher during the video condition than during the live condition. Finally, individual traits did not seem to be related to the participants’ behaviour.

In accordance with our hypotheses, participants did look more often and longer to the confederate when presented in the video on the screen than when present live in the same room. This is in line with previous research by Laidlaw et al. (2011)^26^. The replication suggests that the original findings are robust and reliable, even in a different country and with a different layout. According to Horn and colleagues^99^, social norms strongly modulate where a person should look at what time in the company of another person. Our results also confirm that people follow strict social rules when in a room with other individual, prohibiting them from direct staring at a stranger. However, social rules do not apply in the same way to a video of a person being displayed in the background, allowing people to look more frequently and longer to videos of other people.

Interestingly, even in the video condition, five participants verbally reported during the debriefing that they did not know if they were allowed to watch the video. Therefore, even in this condition, there was uncertainty about the rules that apply according to the experiment, but this uncertainty did not cause the same avoidance as the social rule. It may be the case that social rules have a stronger effect on gaze suppression than uncertain rules due to an ongoing experiment. Or, participants may have forgotten that they were part of an experiment and wearing a mobile eye-tracker when no attention was pulled to this fact^100^, and the content they watched considered neutral to them^101^. This may particularly have been the case in the current study, as the waiting room situation was not believed to be part of the experiment. Therefore, they did not consciously monitor where they looked as much, unless social norms and the chance of interaction applied, as in the live condition. This idea fits with the observation that three participants in the live condition used their phones at some point in the waiting room, despite having previously been told that the eye-tracker was already on and despite being explicitly instructed by the experimenter that they should leave their phones in the preparation room. One of the participants even snatched his phone from the compartment where he was instructed to store it behind the experimenter’s back without her noticing (even though this was, of course, filmed by the eye-tracker which the participant knew was recording his gaze). Therefore, we believe that the presence of the mobile eye-tracker did not affect their social behaviour.

In contrast to our previous hypotheses, the emotions that the confederate displayed did not reliably modulate participants’ attention to her in both live and video condition. This finding was somewhat surprising, as in previous computer-based experiments emotional pictures were preferentially processed^39, 40^ with a preference to look at happy faces^41^. Also, gaze-following during a real life situation was affected by emotional expressions^45^, we therefore also expected emotion effects in the current study. It is possible that in a waiting room situation, emotional expressions in reaction to filling in questionnaires, do not seem to matter sufficiently to people to the point of changing their level to attention to the confederate. In other words, neither when the participant could see someone’s emotional expression in person, nor when they saw the video of the confederate, did they think positive or negative expressions mattered more than a neutral expression. Social rules^16, 17, 99^, and the chance of interaction^26, 27^, can explain the difference in gaze behaviour observed in the live and video conditions, but not the lack of modulation by emotional expression. In the live condition, participants may not have reacted to the emotional expressions because they rarely ever looked at the confederate, independent of her emotional expression. However, there was also no effect of expression when participants looked at the confederate for some time in the video condition. This might be explained by the fact that social content and low-level visual features modulate gaze behaviour to dynamic scenes more than emotional content^102^. However, we expected that people pay more attention to emotions when it is relevant to understand the scene, for example in a movie^103^. Emotional expressions were however not ignored by the participants, which could be seen in one participant explicitly asking the confederate why she was showing a positive expression in the live condition. Interestingly, although our preregistered mixed ANOVAs did not show significant main effects or interactions with the expression of the confederate, when computing linear mixed model to predict the number of direct looks to the confederate based on SIAS as an individual trait, participants looked more often to positive compared to negative expressions of the confederate during the live condition than during the video condition. It is unclear why this effect occurs in the mixed model but not in the mixed ANOVA. It is possible that computational differences between the two statistical methods lead to diverging findings. It may also be the case that the effect is rather small and/or fragile and therefore only appears in mixed linear models. As several measures indicate that there might be a small effect of expression, but none of the preregistered measures reliably confirm it, future research could investigate potential effects in a larger sample. Based on evolutionary theories, emotional content should be preferentially processed because it is potentially relevant for survival^34, 35^. However, in the current study, the waiting room situation was a neutral and safe context, posing no threat to any participants’ life. When the confederate looked annoyed about the questionnaire, this was no realistic threat to the participant and if the confederate looked happy, this had no advantage to the participant. It was therefore not worth to break the social rules to look more at one expression. A different and more robust pattern of gaze may be observed in a situation in which the emotional expression of the other person has a personal relevance to the participants (e.g., an angry person with a weapon who could harm them or a happy person distributing rewards to others, which they could benefit from).

A different gaze pattern could also occur in a situation where an interaction is more common. In the age range (young adults) and western society (German) we studied, it is common sense to avoid conversation when in a waiting room, and only greet an individual upon entrance in the room, but avoid him/her afterwards. It is possible that people only pay attention to a certain type of emotion in situations where an interaction is common^18, 19, 104^. In such situations, people need to check whether the person seems friendly or not, or which emotions they show^20, 21^ to prepare for the upcoming interaction. Interestingly, even when participants did interact with the confederate, it happened not only during the positive expression (confederate smiling), which usually induces approach behaviour^64, 65^, but also during the neutral expression. No interactions during the negative expression occurred, which is in line with studies showing that negative expressions induce avoidance behavior^71–73^. Despite not having many observations, it is plausible that the individual level of extraversion or mood could influence the willingness to interact with another person, in a situation in which this does not commonly happen. To note, a female participant interacted with the confederate during the neutral expression condition and a male participant during the display of positive expression. Another two failed interaction attempts coming from two male participants with the confederate happened during the expression of positive expression. This could indicate a gender effect, in which men might try to interact more with women when they display some “openness” to interaction^105^. Considering we did not have enough events to test this and it was not our main objective, those observations are only speculative.

Regarding individual differences, we did not find gaze behaviour in the waiting room paradigm to relate to autistic traits nor social anxiety. This replicates the finding by Horn and colleagues who showed that autistic traits and social anxiety traits did not relate to gaze behaviour to a confederate either just working at the computer, talking on the phone or wearing headphone while working in front of a computer^99^. Similarly, studies in naturalistic social scenarios demonstrated that the looking behaviour of people with high autistic traits do not differ from those with lower autistic traits when interacting with a experimenter in face-to-face interactions^106, 107^. Social anxiety traits also did not correlate with visual attention, but it did affect physiological measures, e.g. heart rate^108^. In addition to those studies, the current study only tested healthy participants, which were at least not formally diagnosed with autism or social anxiety. Patients diagnosed with either disorder might display a different behaviour, as currently under investigation^109^. Further studies are needed in both cases.

In this study, we also investigated potential covert attention to the confederate, by defining instances when participants looked in the direction, but not directly at the confederate (see Fig. 3, for the areas we considered as possible covert looks). It is important to note that this procedure does not guarantee that the participant was attending covertly to the confederate. Alternatively, they might simply be attending in the direction of their gaze.

Participants looked longer and more frequently at the area around the face and upper body of the confederate, indicating covert attention, during the video condition than during the live condition. Although this contrasts our initial hypothesis, it is in line with Laidlaw and colleagues’ research^26^. In their study, participants turned their head but did not fixate the confederate more frequently during the video condition, than during the live condition. One explanation is that participants look more to the video than the live confederate overall and therefore show more instances of gaze around the area of the video confederate. Due to measurement inaccuracy, this could lead to distributions of fixation around the face of the confederate. Alternatively, participants might not need to make large eye movements to covertly attend to her, as they could possibly more efficiently pay covert attention. However, in contrast to the current work, Dosso and Hyuhn^30^ showed that when people intend to pay covert attention to someone, they did it more when the other person was physically present and payed overt attention to the video of the same person. Altogether, this indicates that indirect looks around the confederate might represent simply the opportunity to look to the video as much as wanted, and only partially an indication of covert attention. On the other hand, in the live condition, even if the participant did not look directly at the confederate, per our definition, a covert attention shift would still involve looking at the close proximity of the confederate. Participants may worry that this close proximity could be misinterpreted in a social situation and subsequently be perceived as awkward or even rude. Therefore, covert looks as defined in our methods based on the previous literature^26^ might show similar patterns as overt orienting. Additional measures, such as combined EEG, may help disentangle covert and overt attention in the waiting room paradigm^110, 111^.

In summary, the current study aimed to investigate effects of social context and emotional expression of a confederate on participants’ gaze in a waiting room situation. Gaze was reliably more often and longer directed at videos than at live confederates, in line with social rules. However, emotional expressions did not reliably influence gaze behaviour in the live or video situation. The observed effects were independent of natural variations in autistic and anxious traits. This suggests that the rules of social contexts very reliably affect gaze behaviour while emotional expression do not lead to such strong effects.

### Supplementary Information

Supplementary Information.

## Data Availability

We shared scripts for power analysis and data analysis via the “Open Science Framework” platform (https://osf.io/wznfj/). We shared the full coded gaze data, all behavioural scripts and videos of the confederate that do not include identifiable participant information via the “Open Science Framework” platform (https://osf.io/wznfj/).
