# Supplementary material for: Effects of emotional content on social inhibition of gaze in live social and non-social situations
Source: Sci Rep. 2023 Aug 29;13:14151. doi: 10.1038/s41598-023-41154-w (PMC10465544; doi:10.1038/s41598-023-41154-w)
Supplement: Supplementary file 1 — Supplementary Information. [file 41598_2023_41154_MOESM1_ESM.pdf]

Effects of emotional content on social inhibition of gaze in live social and non-social situations

Laura Pasqualette[1], Louisa Kulke[1*]

[1] Neurocognitive Developmental Psychology, Friedrich-Alexander University Erlangen - Nürnberg, Erlangen, Germany

* Corresponding author: Louisa Kulke (louisa.kulke@fau.de).

Sample Size Calculation

Laura Pasqualette, Louisa Kulke

Set seed for reproducibility

```
set.seed(1010)
```

Calculating sample size for study

We have a 2 (between-subject: social vs non-social) x 3 (within-subject: negative vs positive vs neutral) design

Tutorial: https://cran.r-project.org/web/packages/paramtest/vignettes/Simulating-Power.html

Calculate sample for two-sample t-test (considering social x non-social conditions)

First determine the Cohen's d from Laidlaw et al. (2011)

Method used: https://www.bwgriffin.com/gsu/courses/edur9131/content/Effect_Sizes_pdf5.pdf

```
N = 26 #total subject number (half per group)
t = 2.97 # t(12.21) = 2.97, info taken from page 5550 (Laidlaw, 2011) from the t-test
  #between looking times [s] to confederate or video-taped recording of confederate
cohensd_fixduration <- round(2*t/sqrt(N-2),2)

t1 = 3.24 #t(12.39) = 3.24, info taken from page 5550 (Laidlaw, 2011) from the t-test
#between number of fixations to confederate or video-taped recording of confederate
cohensd_fixtimes <- round(2*t1/sqrt(N-2), 2)
```

```
#n = number in each group
pwr.t.test(power = 0.95, d= 1.21, type='two.sample') # Similar Cohen's d to Laidlaw et al (2011)
```

```
##
##      Two-sample t test power calculation
##
##               n = 18.76896
##               d = 1.21
##       sig.level = 0.05
##           power = 0.95
##     alternative = two.sided
##
## NOTE: n is number in *each* group
```

```
pwr.t.test(power = 0.95, d= 1.32, type='two.sample') # Similar Cohen's d to Laidlaw et al (2011)
```

```
##
##      Two-sample t test power calculation
##
##               n = 15.94443
##               d = 1.32
##       sig.level = 0.05
##           power = 0.95
##     alternative = two.sided
##
## NOTE: n is number in *each* group
```

Generate data to run multiple T-test (two-sided)

First, we created a user-defined function

```
# create user-defined function to generate and analyze data
t_func <- function(simNum, N, d) {
  Social <- rnorm(N, 0, 1)
  NSocial <- rnorm(N, d, 1)

  t <- t.test(Social, NSocial, var.equal=TRUE)  # run t-test on generated data
  stat <- t$statistic
  p <- t$p.value

  return(c(t=stat, p=p, sig=(p < .05)))
  # return a named vector with the results we want to keep
}
```

Varying N and Cohen's d

Run power test for different sample sizes and different Cohen's d. Additionally, variation of expected t-values and p-values are calculated.

```
# run analysis
power_ttest_vary2 <- grid_search(t_func, params=list(N=c(10, 20,30,40,50,60,
                                                        70, 80, 90, 100),
                                                   d=c(cohensd_fixduration, cohensd_fixtimes)),
                                 n.iter=5000, output='data.frame')
```

```
## Running 100,000 tests...
```

```
power2 <- results(power_ttest_vary2) %>%
  group_by(N.test, d.test) %>%
  summarise(power=mean(sig), t_value = mean(t.t), p_value = mean(p))
```

```
## `summarise()` has grouped output by 'N.test'. You can override using the `.groups` argument.
```

```
print(power2)
```

```
## # A tibble: 20 x 5
## # Groups:   N.test [10]
##    N.test d.test power t_value  p_value
##     <dbl>  <dbl> <dbl>   <dbl>    <dbl>
##  1     10   1.21 0.725   -2.82 6.29e- 2
##  2     10   1.32 0.791   -3.07 4.39e- 2
##  3     20   1.21 0.961   -3.94 7.93e- 3
##  4     20   1.32 0.977   -4.23 4.93e- 3
##  5     30   1.21 0.998   -4.76 9.22e- 4
##  6     30   1.32 0.998   -5.20 4.93e- 4
##  7     40   1.21 0.999   -5.47 1.99e- 4
##  8     40   1.32 1.00    -5.94 6.17e- 5
##  9     50   1.21 1       -6.09 3.59e- 5
## 10     50   1.32 1       -6.68 7.48e- 6
## 11     60   1.21 1       -6.64 4.38e- 6
## 12     60   1.32 1       -7.29 8.86e- 7
## 13     70   1.21 1       -7.22 5.11e- 7
## 14     70   1.32 1       -7.81 4.36e- 8
## 15     80   1.21 1       -7.68 7.15e- 8
## 16     80   1.32 1       -8.39 4.51e- 9
## 17     90   1.21 1       -8.14 2.14e- 8
## 18     90   1.32 1       -8.85 8.56e-10
## 19    100   1.21 1       -8.61 3.24e- 9
## 20    100   1.32 1       -9.37 3.79e-11
```

Results

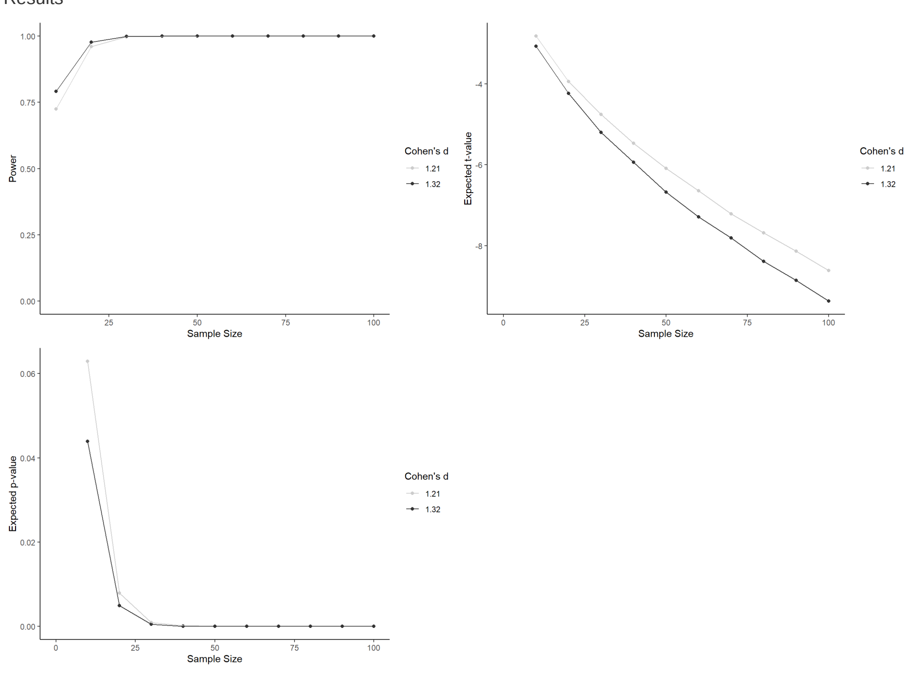

Sample calculation for a mixed ANOVA

Calculating sample size using the equivalent Cohen's f to the previous known Cohen's d (1.21/2 = 0.605), power = 0.95 and alpha error = 0.05.

```
# ng (number of groups) = 2
# nm (number of measures) = 3
# nscor (nonsphericity correction) = Nonsphericity correction coefficient. The nonsphericity correction coefficie
nt is a measure of the degree of sphericity in the population. A coefficient of 1 means sphericity is met, while
 a coefficient less than 1 means not met. The smaller value of the coefficient means the further departure from s
phericity. The lowest value of the coefficient is 1/(nm-1) where nm is the total number of measurements. Two viab
le approaches for computing the empirical nonsphericity correction coefficient are sggested. One is by Greenhouse
and Geisser (1959), the other is by Huynh and Feldt (1976).

wp.rmanova(f = 0.605, ng = 2, nm = 3, nscor = 1, alpha = 0.05, power = 0.95, type =0) # between-subject effect
```

```
## Repeated-measures ANOVA analysis
##
##            n     f ng nm nscor alpha power
##     37.53791 0.605  2  3     1  0.05  0.95
##
## NOTE: Power analysis for between-effect test
## URL: http://psychstat.org/rmanova
```

```
wp.rmanova(f = 0.605, ng = 2, nm = 3, nscor = 1, alpha = 0.05, power = 0.95, type =1) # within-subject effect
```

```
## Repeated-measures ANOVA analysis
##
##            n     f ng nm nscor alpha power
##     43.74916 0.605  2  3     1  0.05  0.95
##
## NOTE: Power analysis for within-effect test
## URL: http://psychstat.org/rmanova
```

```
wp.rmanova(f = 0.605, ng = 2, nm = 3, nscor = 1, alpha = 0.05, power = 0.95, type =2) # interaction effect
```

```
## Repeated-measures ANOVA analysis
##
##            n     f ng nm nscor alpha power
##     43.74916 0.605  2  3     1  0.05  0.95
##
## NOTE: Power analysis for interaction-effect test
## URL: http://psychstat.org/rmanova
```

```
#n represent the TOTAL sample!
```

Simulating data for mixed ANOVA

Generating data (normally distributed) for each condition

Extracting difference between means for the between-subject condition (social vs non-social): taken from Laidlaw (2011) - based on values from Fig 2 - head turn, fixation on confederate

Used the website to infer the values from the figure: https://apps.automeris.io/wpd/

Type of data used to: Mean looking time at the confederate (in seconds)

Mean from live condition: 0.87 (our reference value)
Mean from the videotaped condition: 14.9

Calculating the standard deviations (SDs) from the same figure

SE = upper part of SE - mean = real SE value
Live: SE = 1.40 - 0.87 = 0.53 –> SD = 0.53 * sqrt(13) = 1.9
Videotaped: SE = 19.7 - 14.9 = 4.8 –> SD = 4.8 * sqrt(13) = 17.3
A Standard Deviation of 1.9 will be used throughout the simulation, as it is coherent with the data used for Mean Fixation Time (s). In the planned study, we will use non-parametric tests if the homogeneity of variance assumption is violated in our sample.

Modelling the difference among means for the within-subject condition (negative vs neutral vs positive expressions):

Taken from Gamble et al. (2010), Figure 1 - proportion of healthy individuals' fixations between happy/neutral and angry/neutral faces. This proportion reflects the relative frequency of looking toward the angry face compared to the neutral face when shown an angry-neutral face pair. We chose those expressions to represent the positive and negative emotional faces.

Proportion of Happy/total number of fixation to the pair of happy and neutral faces = 0.55
Proportion of Angry/total number of fixation to the pair of angry and neutral faces = 0.47

From these proportions we can estimate which percentage belongs to the neutral expression. E.g., we consider the means from Laidlaw et al. as the 45% in the positive-neutral expression pair. From this we compute how much 100% is, and then the corresponding value for positive face (55%).

Calculating a mean for positive expression in social condition:
(0.87/45) * 100 = 100% is 1.93
1.93 * 0.55 = 1.06

Calculating a mean for negative expression in social condition:
(0.87/53) * 100 = 1.64
1.64 * 0.47 = 0.77

Calculating a mean for positive expression in non-social condition:
(14.9/45) * 100 = 100% is 33.1
33.1 * 0.55 = 18.2

Calculating a mean for negative expression in non-social condition:
(14.9/53) * 100 = 28.1
28.1 * 0.47 = 13.2

Simulation to calculate required sample size for a sufficiently large effect size:

```
#create user-defined function to generate and analyze data
# means used as above
aov_func <- function(simNum, N, SD) {


  DataSocial <- data.frame(Social_positive = rnorm(N, 1.06, SD),
                        Social_neutral= rnorm(N, 0.87, SD), #0.87 (value for fixations in social condition from La
idlaw as basis)
                        Social_negative = rnorm(N, 0.77, SD),
                        Subject = 1:N)


  DataSocial%>% #Organize data
    gather(key = Sociality, value = MeanFix, starts_with("Social")) %>%
    separate(Sociality, c("Sociality", "Expression"), sep = '_')%>%
    mutate(Sociality = as.factor(Sociality), Expression = as.factor(Expression)) -> DS


  # Data for non-social Condition
  DataNSocial <- data.frame(NSocial_positive = rnorm(N, 18.2, SD),
                         NSocial_neutral= rnorm(N, 14.9, SD),  # 14.9 (value for the videotaped condition by Laidl
aw)
                         NSocial_negative = rnorm(N, 13.2, SD),
                         Subject = N+1:N)

  DataNSocial%>% # organize data
    gather(key = Sociality, value = MeanFix, starts_with("Nsocial")) %>%
    separate(Sociality, c("Sociality", "Expression"), sep = '_') %>%
    mutate(Sociality = as.factor(Sociality), Expression = as.factor(Expression))-> DNS

  # Bind both tables together by their rows
  SocialFinal <- bind_rows(DS, DNS)
  SocialFinal %>%
    group_by(Subject, Sociality, Expression)

  as.data.frame(SocialFinal) -> SocialFinal

  res.aov <- anova_test(data = SocialFinal, dv = MeanFix, wid = Subject,
                         between = Sociality, within = Expression, effect.size = "pes")  # run mixed anova on gene
rated data
  F_Sociality <- res.aov$ANOVA$F[1]
  F_Expression <- res.aov$ANOVA$F[2]
  F_Soc_Exp <- res.aov$ANOVA$F[3]

  p_Sociality <- res.aov$ANOVA$p[1]
  p_Expression <- res.aov$ANOVA$p[2]
  p_Soc_Exp <- res.aov$ANOVA$p[3]

  pes_Sociality <- res.aov$ANOVA$pes[1]
  pes_Expression <- res.aov$ANOVA$pes[2]
  pes_Soc_Exp <- res.aov$ANOVA$pes[3]

  return(c(F_Sociality=F_Sociality, F_Expression = F_Expression,
           F_Soc_Exp= F_Soc_Exp, p_Sociality=p_Sociality, p_Expression = p_Expression,
           p_Soc_Exp= p_Soc_Exp, pes_Sociality=pes_Sociality, pes_Expression = pes_Expression,
           pes_Soc_Exp= pes_Soc_Exp))
  # return a named vector with the results we want to keep
}
```

We set different sample sizes as an example and we decided to use 1000 iterations

```
power_ttest_vary <- grid_search(aov_func, params=list(N=c(10,20,24,30,40,50, 60, 70, 80, 90, 100, 110, 120, 130,
140, 150),
                                                   SD=1.9),
                              n.iter=1000, output='data.frame', parallel='snow')
```

```
## Running 16,000 tests...
```

```
#Extract the required information
power <- results(power_ttest_vary) %>%
  group_by(N.test, SD.test) %>%
  summarise(F_Sociality=mean(F_Sociality), F_Expression = mean(F_Expression),
            F_Soc_Exp= mean(F_Soc_Exp), p_Sociality=mean(p_Sociality), p_Expression = mean(p_Expression),
            p_Soc_Exp= mean(p_Soc_Exp), pes_Sociality=mean(pes_Sociality), pes_Expression = mean(pes_Expression),
            pes_Soc_Exp= mean(pes_Soc_Exp))
```

```
## `summarise()` has grouped output by 'N.test'. You can override using the `.groups` argument.
```

```
print(power)
```

```
## # A tibble: 16 x 11
## # Groups:   N.test [16]
##    N.test SD.test F_Sociality F_Expression F_Soc_Exp p_Sociality p_Expression
##     <dbl>   <dbl>       <dbl>        <dbl>     <dbl>       <dbl>        <dbl>
##  1     10     1.9        982.         11.3      9.25   2.46e- 15     5.43e- 3
##  2     20     1.9       1840.         21.8     17.7    5.75e- 30     3.79e- 5
##  3     24     1.9       2187.         25.9     20.5    1.35e- 34     3.19e- 6
##  4     30     1.9       2730.         31.8     25.3    1.87e- 44     1.18e- 6
##  5     40     1.9       3609.         41.1     33.3    1.25e- 61     2.62e- 9
##  6     50     1.9       4493.         51.5     41.4    2.68e- 77     8.52e-10
##  7     60     1.9       5367.         61.7     49.3    4.80e- 93     8.44e-14
##  8     70     1.9       6220.         71.8     56.9    8.26e-109     2.92e-15
##  9     80     1.9       7129.         81.6     65.0    9.03e-124     5.80e-21
## 10     90     1.9       8006.         91.1     72.7    1.35e-141     1.55e-22
## 11    100     1.9       8846.        102.      80.8    2.58e-155     2.80e-26
## 12    110     1.9       9722.        112.      88.8    2.83e-171     4.95e-27
## 13    120     1.9      10627.        121.      96.4    5.33e-189     9.27e-29
## 14    130     1.9      11513.        132.     105.     5.64e-205     4.46e-33
## 15    140     1.9      12415.        142.     113.     9.82e-220     4.80e-34
## 16    150     1.9      13235.        153.     121.     3.06e-235     9.54e-39
## # ... with 4 more variables: p_Soc_Exp <dbl>, pes_Sociality <dbl>,
## #   pes_Expression <dbl>, pes_Soc_Exp <dbl>
```

Calculating Cohen's f from partial eta-squared: f = sqr(eta^2 / (1 - eta^2))

```
#Extract the required information
power_v2 <- power %>%
  mutate(Cohensf_Sociality = sqrt(
    (pes_Sociality)/(1-pes_Sociality))) %>%
  mutate(Cohensf_Expression = sqrt(
    (pes_Expression)/(1-pes_Expression))) %>%
  mutate(Cohensf_Soc_Exp = sqrt(
    (pes_Soc_Exp)/(1-pes_Soc_Exp)))
print(power_v2)
```

```
## # A tibble: 16 x 14
## # Groups:   N.test [16]
##    N.test SD.test F_Sociality F_Expression F_Soc_Exp p_Sociality p_Expression
##     <dbl>   <dbl>       <dbl>        <dbl>     <dbl>       <dbl>        <dbl>
##  1     10     1.9        982.         11.3      9.25   2.46e- 15     5.43e- 3
##  2     20     1.9       1840.         21.8     17.7    5.75e- 30     3.79e- 5
##  3     24     1.9       2187.         25.9     20.5    1.35e- 34     3.19e- 6
##  4     30     1.9       2730.         31.8     25.3    1.87e- 44     1.18e- 6
##  5     40     1.9       3609.         41.1     33.3    1.25e- 61     2.62e- 9
##  6     50     1.9       4493.         51.5     41.4    2.68e- 77     8.52e-10
##  7     60     1.9       5367.         61.7     49.3    4.80e- 93     8.44e-14
##  8     70     1.9       6220.         71.8     56.9    8.26e-109     2.92e-15
##  9     80     1.9       7129.         81.6     65.0    9.03e-124     5.80e-21
## 10     90     1.9       8006.         91.1     72.7    1.35e-141     1.55e-22
## 11    100     1.9       8846.        102.      80.8    2.58e-155     2.80e-26
## 12    110     1.9       9722.        112.      88.8    2.83e-171     4.95e-27
## 13    120     1.9      10627.        121.      96.4    5.33e-189     9.27e-29
## 14    130     1.9      11513.        132.     105.     5.64e-205     4.46e-33
## 15    140     1.9      12415.        142.     113.     9.82e-220     4.80e-34
## 16    150     1.9      13235.        153.     121.     3.06e-235     9.54e-39
## # ... with 7 more variables: p_Soc_Exp <dbl>, pes_Sociality <dbl>,
## #   pes_Expression <dbl>, pes_Soc_Exp <dbl>, Cohensf_Sociality <dbl>,
## #   Cohensf_Expression <dbl>, Cohensf_Soc_Exp <dbl>
```

Create plots for F, p, Cohen's f, and partial eta-squared (pes) for Sociality Effects on Fixation Time across different sample sizes

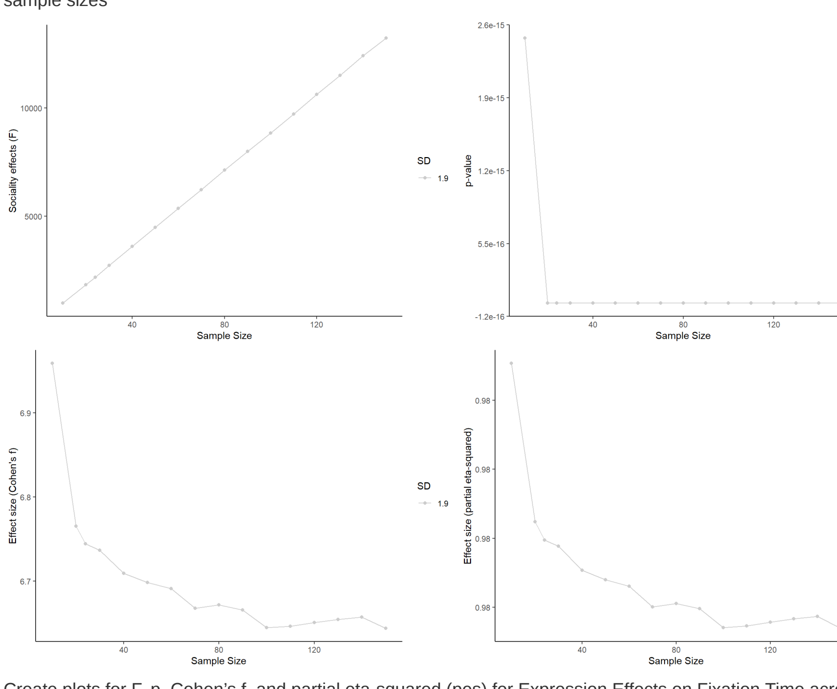

Create plots for F, p, Cohen's f, and partial eta-squared (pes) for Expression Effects on Fixation Time across different sample sizes

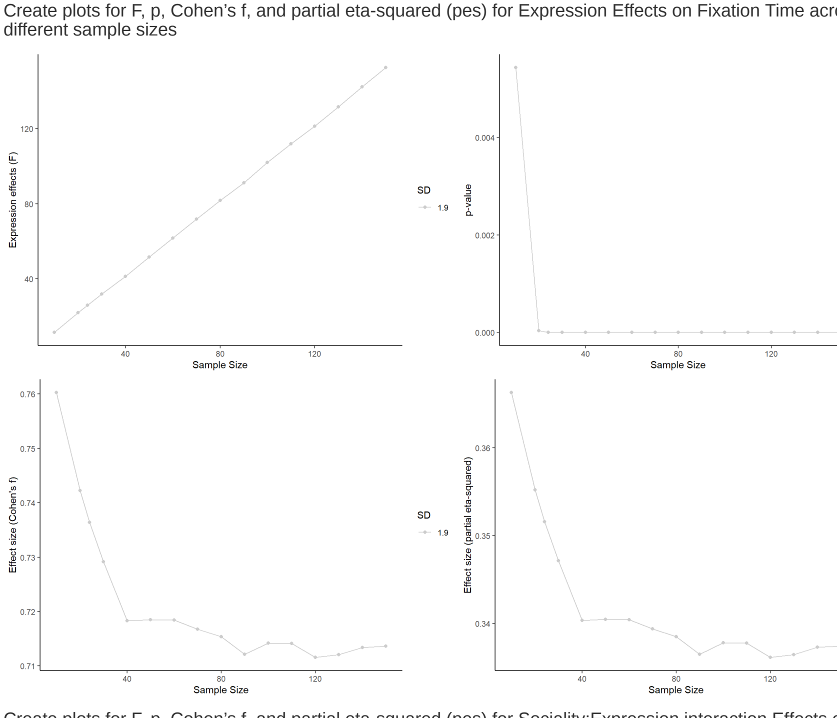

Create plots for F, p, Cohen's f, and partial eta-squared (pes) for Sociality:Expression interaction Effects on Fixation Time across different sample sizes

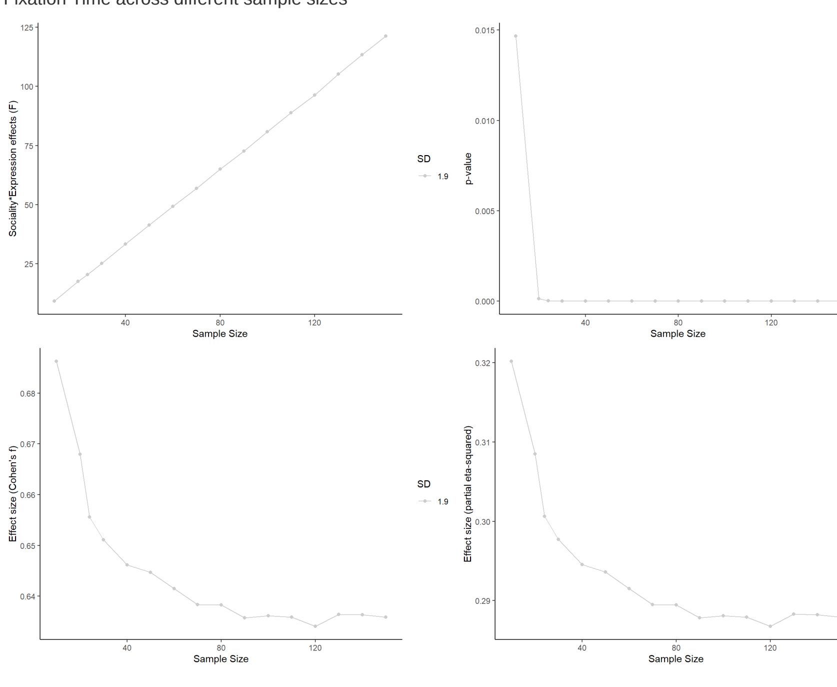

References

```
cite_packages()
```


```
##   - Alboukadel Kassambara (2021). rstatix: Pipe-Friendly Framework for Basic Statistical Tests. R package vers
ion 0.7.0. https://CRAN.R-project.org/package=rstatix
##   - Douglas Bates and Martin Maechler (2021). Matrix: Sparse and Dense Matrix Classes and Methods. R package v
ersion 1.3-2. https://CRAN.R-project.org/package=Matrix
##   - Douglas Bates, Martin Maechler, Ben Bolker, Steve Walker (2015). Fitting Linear Mixed-Effects Models Using
lme4. Journal of Statistical Software, 67(1), 1-48. doi:10.18637/jss.v067.i01.
##   - H. Wickham. ggplot2: Elegant Graphics for Data Analysis. Springer-Verlag New York, 2016.
##   - Hadley Wickham (2019). stringr: Simple, Consistent Wrappers for Common String Operations. R package versio
n 1.4.0. https://CRAN.R-project.org/package=stringr
##   - Hadley Wickham (2021). forcats: Tools for Working with Categorical Variables (Factors). R package version
0.5.1. https://CRAN.R-project.org/package=forcats
##   - Hadley Wickham (2021). tidyr: Tidy Messy Data. R package version 1.1.3. https://CRAN.R-project.org/package
=tidyr
##   - Hadley Wickham and Jim Hester (2020). readr: Read Rectangular Text Data. R package version 1.4.0. https://
CRAN.R-project.org/package=readr
##   - Hadley Wickham, Romain François, Lionel Henry and Kirill Müller (2021). dplyr: A Grammar of Data Manipulat
ion. R package version 1.0.5. https://CRAN.R-project.org/package=dplyr
##   - Jeffrey Hughes (2017). paramtest: Run a Function Iteratively While Varying Parameters. R package version
0.1.0. https://CRAN.R-project.org/package=paramtest
##   - JJ Allaire and Yihui Xie and Jonathan McPherson and Javier Luraschi and Kevin Ushey and Aron Atkins and Ha
dley Wickham and Joe Cheng and Winston Chang and Richard Iannone (2021). rmarkdown: Dynamic Documents for R. R pa
ckage version 2.7. URL https://rmarkdown.rstudio.com.
##   - Kirill Müller and Hadley Wickham (2021). tibble: Simple Data Frames. R package version 3.1.0. https://CRA
N.R-project.org/package=tibble
##   - Lionel Henry and Hadley Wickham (2020). purrr: Functional Programming Tools. R package version 0.3.4. http
s://CRAN.R-project.org/package=purrr
##   - Makowski, D., Ben-Shachar, M.S., Patil, I. & Lüdecke, D. (2020). Automated reporting as a practical tool t
o improve reproducibility and methodological best practices adoption. CRAN. Available from https://github.com/eas
ystats/report. doi: .
##   - Martin Becker and Stefan Klößner (2017). PearsonDS: Pearson Distribution System. R package version 1.1. ht
tps://CRAN.R-project.org/package=PearsonDS
##   - R Core Team (2021). R: A language and environment for statistical computing. R Foundation for Statistical
Computing, Vienna, Austria. URL https://www.R-project.org/.
##   - Stephane Champely (2020). pwr: Basic Functions for Power Analysis. R package version 1.3-0. https://CRAN.R
-project.org/package=pwr
##   - Venables, W. N. & Ripley, B. D. (2002) Modern Applied Statistics with S. Fourth Edition. Springer, New Yor
k. ISBN 0-387-95457-0
##   - Wickham et al., (2019). Welcome to the tidyverse. Journal of Open Source Software, 4(43), 1686, https://do
i.org/10.21105/joss.01686
##   - Yves Rosseel (2012). lavaan: An R Package for Structural Equation Modeling. Journal of Statistical Softwar
e, 48(2), 1-36. URL https://www.jstatsoft.org/v48/i02/.
##   - Zhiyong Zhang and Yujiao Mai (2018). WebPower: Basic and Advanced Statistical Power Analysis. R package ve
rsion 0.5.2. https://CRAN.R-project.org/package=WebPower
```

Supplementary Material

Behavioural script for the confederate and experimenter's actions

Social condition and video condition

Experimenter:

Upon the arrival of the participant, the experimenter will briefly explain the aims of the study i.e. in this moment, the decoy task. The experimenter will tell the participants that they will wear an eye-tracker to monitor their eye-movements while they see some photographs and paintings in the experiment room. The experimenter will ask the participant to sign a consent form on a clipboard.

Subsequently, the participant will be asked to store his/her belongings (e.g. bags, phone, etc.) in a safe location, and they will be asked if they wish to use the toilet before the experiment starts.

Afterwards, the experimenter will setup the eye-tracking glasses on the participant and attach a pouch around the participant's waist, containing the gaze recording phone. Next, the experimenter will state that they need to finish setting up the experiment in the other room, so they will guide the participant to the waiting room, and ask them to have a seat at a designated chair and wait (in both the social and video conditions).

After three minutes, the experimenter will go back into the waiting room to pick up and instruct the participant to freely view different photos and paintings hanging in the adjacent room. In the video condition, in case the participant asks about the content of the video of the confederate, the experimenter will say that it belongs to an experiment from the previous year and that it is currently being analyzed by another researcher.

The experimenter will tell the participant that he or she will wait in the other room for the participant to finish the task.

After completing the decoy task, the participant will be led back to the waiting room to fill in the necessary questionnaires. At this moment, neither the confederate (social condition) nor the video on the monitor (video condition) will be present in the room. After he/she finishes filling out the questionnaires, the experimenter will then orally ask the questions in the debriefing questionnaire and write the participant's answers. Finally, the experimenter will debrief the participant about the true aim of the study and ask him/her to sign a second written consent. Participants will be reminded of the option to have their data deleted during debriefing.

Social Condition

Confederate:

As the participant enters the waiting room, the confederate will be already there, sitting in the designated chair, pretending to be another participant filling out some questionnaires on a clipboard.

The confederate will look at the direction of the participant and nonverbally acknowledge his/her presence by nodding with a friendly expression. Afterwards, the confederate will start to pretend to fill out the questionnaires again and will not look at the participant anymore. As soon as the experimenter leaves the room and closes the door, the timing of the first condition will start.

The confederate will fill out a questionnaire of three pages on the clipboard. On each page, two pieces of information will be written within a larger text: at the beginning of the page, the expressions the confederate should make (i.e. negative, neutral or positive). The confederate is blind to the order of expressions and shows this expression in response to reading it. After half of the page, the text informs the confederate to look up briefly. While the confederate looks up, he/she will continue to display the assigned expression.

Each expression will be performed for a minute, and by turning the page, the confederate will change to the next expression. The whole performance of the confederate will total 3 minutes.

If the participant tries to interact verbally (e.g. start a conversation) with the confederate at any point of those three minutes, the confederate will respond to the participant, to simulate a normal situation in a waiting room.

The confederate will not signal any farewell to the participant, unless they had engaged in conversation previously.

Analysis Script

Laura Pasqualette, Louisa Kulke

Calculating demographic information

```
#opening demographic data
Demographic <- read.csv2(file = 'demographic.csv', sep=";", header=TRUE)
Demographic <- Demographic %>% select_if(~!(all(is.na(.)) | all(. == ""))) %>% mutate(Gender = as.factor(Gender))
%>% mutate(Type.of.Instruction = as.factor(Type.of.Instruction)) %>% mutate(Participant.ID = as.factor(Participant.ID)) %>% arrange(Participant.ID)

mean_age = mean(Demographic$Age) #calculate the average age of participants
sd_age = sd(Demographic$Age) # calculate the standard deviation for the ages

gender = summary(Demographic$Gender) # count the number of men,women and diverse in the experiment
ratio_gender = 40/(7+40+1) # calculate ratio for gender
```

Investigate model assumptions

```
### Checking assumptions for mixed ANOVA

#open all the relevant data
#First Look Latency
FirstLookLatency <- read.csv2(file = 'FirstLookLatency.csv', sep=";", header=TRUE)
FirstLookLatency <- FirstLookLatency %>% select_if(~!(all(is.na(.)) | all(. == ""))) %>% mutate(Participant.ID =
as.factor(Participant.ID)) %>% mutate(Type.of.Instruction = as.factor(Type.of.Instruction)) %>%
  mutate(Emotion.Displayed = as.factor(Emotion.Displayed))
#testing without outliers - same results
FirstLookLatency %>% filter(Participant.ID != c("LP21")) -> FirstLookLatency
#Number of direct looks to the confederate
Number.Looks <- read.csv2(file = 'number_looks.csv', sep=",", header=TRUE)
Number.Looks <- Number.Looks %>% mutate(Participant.ID = as.factor(Participant.ID)) %>% mutate(Condition = as.factor(Condition)) %>%
  mutate(Emotion.Displayed = as.factor(Emotion.Displayed))
Number.Looks$Emotion.Displayed <- recode_factor(Number.Looks$Emotion.Displayed , "Negatve" = "Negative")
#testing without outliers - same results
Number.Looks %>% filter(Participant.ID != c("LP21")) -> Number.Looks
#Proportion of Looks to Face and Upper body
Proportion.Looks <- read.csv2(file = 'look_proportion.csv', sep=",", header=TRUE)
Proportion.Looks <- Proportion.Looks %>% mutate(Participant.ID = as.factor(Participant.ID)) %>% mutate(Condition
= as.factor(Condition)) %>% mutate(Emotion.Displayed = as.factor(Emotion.Displayed)) %>% mutate(Proportion.Looks
= as.numeric(Proportion.Looks))
Proportion.Looks$Emotion.Displayed <- recode_factor(Proportion.Looks$Emotion.Displayed , "Negatve" = "Negative")
#testing without outliers - same results
Proportion.Looks %>% filter(Participant.ID != c("LP21")) -> Proportion.Looks
#Number of Covert Looks
Covert.Number <- read.csv2(file = 'Covert_number_looks.csv', sep=",", header=TRUE)
Covert.Number <- Covert.Number %>% mutate(Participant.ID = as.factor(Participant.ID)) %>% mutate(Condition = as.factor(Condition)) %>% mutate(Emotion.Displayed = as.factor(Emotion.Displayed))
Covert.Number$Emotion.Displayed <- recode_factor(Covert.Number$Emotion.Displayed , "Negatve" = "Negative")
#testing without outliers - same results
Covert.Number %>% filter(Participant.ID != c("LP21")) -> Covert.Number
#Duration of Covert Looks
Covert.Duration <- read.csv2(file = 'Covert_look_duration.csv', sep=",", header=TRUE)
Covert.Duration <- Covert.Duration %>% mutate(Participant.ID = as.factor(Participant.ID)) %>% mutate(Condition =
as.factor(Condition)) %>% mutate(Emotion.Displayed = as.factor(Emotion.Displayed))
Covert.Duration$Emotion.Displayed <- recode_factor(Covert.Duration$Emotion.Displayed , "Negatve" = "Negative")
#testing without outliers - same results2
Covert.Duration %>% filter(Participant.ID != c("LP21")) -> Covert.Duration


# In general: check outliers on the data - There are outliers, but those are not wrong data, only different types
of behavior
# First look Latency
FirstLookLatency %>%
  group_by(Type.of.Instruction, Emotion.Displayed) %>%
  identify_outliers(FirstLookLatency)
#Number of direct looks
Number.Looks %>%
  group_by(Condition, Emotion.Displayed) %>%
  identify_outliers(Number.Looks)
#Proportion of looks
Proportion.Looks %>%
  group_by(Condition, Emotion.Displayed) %>%
  identify_outliers(Proportion.Looks)
#Covert number of looks
Covert.Number %>%
  group_by(Condition, Emotion.Displayed) %>%
  identify_outliers(Number.Looks)
#Covert duration of looks
Covert.Duration %>%
  group_by(Condition, Emotion.Displayed) %>%
  identify_outliers(Duration.Looks)

# Check normality of raw data per condition

# First look Latency
FirstLookLatency %>%
  group_by(Type.of.Instruction, Emotion.Displayed) %>%
  shapiro_test(FirstLookLatency)
#Number of direct looks
Number.Looks %>%
  group_by(Condition, Emotion.Displayed) %>%
  shapiro_test(Number.Looks)
#Proportion of looks
Proportion.Looks %>%
  group_by(Condition, Emotion.Displayed) %>%
  shapiro_test(Proportion.Looks)
#Covert number of looks
Covert.Number %>%
  group_by(Condition, Emotion.Displayed) %>%
  shapiro_test(Number.Looks)
#Covert duration of looks
Covert.Duration %>%
  group_by(Condition, Emotion.Displayed) %>%
  shapiro_test(Duration.Looks)


# Create a QQ plot to visualize

# First look Latency
ggqqplot(FirstLookLatency, "FirstLookLatency", ggtheme = theme_bw()) +
  facet_grid(Type.of.Instruction ~ Emotion.Displayed)
#Number of direct looks
ggqqplot(Number.Looks, "Number.Looks", ggtheme = theme_bw()) +
  facet_grid(Condition ~ Emotion.Displayed)
#Proportion of looks
ggqqplot(Proportion.Looks, "Proportion.Looks", ggtheme = theme_bw()) +
  facet_grid(Condition ~ Emotion.Displayed)
#Covert number of looks
ggqqplot(Covert.Number, "Number.Looks", ggtheme = theme_bw()) +
  facet_grid(Condition ~ Emotion.Displayed)
#Covert duration of looks
ggqqplot(Covert.Duration, "Duration.Looks", ggtheme = theme_bw()) +
  facet_grid(Condition ~ Emotion.Displayed)


# Check homogeneity of variance assumption
# First look Latency
FirstLookLatency %>%
  group_by(Emotion.Displayed) %>%
  levene_test(FirstLookLatency ~ Type.of.Instruction)
#Number of direct looks
Number.Looks %>%
  group_by(Emotion.Displayed) %>%
  levene_test(Number.Looks ~ Condition)
#Proportion of looks
Proportion.Looks %>%
  group_by(Emotion.Displayed) %>%
  levene_test(Proportion.Looks ~ Condition)
#Covert number of looks
Covert.Number %>%
  group_by(Emotion.Displayed) %>%
  levene_test(Number.Looks ~ Condition)
#Covert duration of looks
Covert.Duration %>%
  group_by(Emotion.Displayed) %>%
  levene_test(Duration.Looks ~ Condition)


### Model for Linear Mixed Model Analysis

# First look Latency - model does not work because there is only one observation per participant, which keeps me
from adding them as a random effect. Therefore, this variable will be excluded from further analysis.

#Number of direct looks
lmm_Number <- lmer(Number.Looks ~ Condition + Emotion.Displayed + (1|Participant.ID), data = Number.Looks)
#Proportion of looks
lmm_Prop <- lmer(Proportion.Looks ~ Condition + Emotion.Displayed + (1|Participant.ID), data = Proportion.Looks)
#Covert number of looks
lmm_Cov_num <- lmer(Number.Looks ~ Condition + Emotion.Displayed + (1|Participant.ID), data = Covert.Number)
#Covert duration of looks
lmm_Cov_dur <- lmer(Duration.Looks ~ Condition + Emotion.Displayed + (1|Participant.ID), data = Covert.Duration)


# Check for residuals normality

check_normality(lmm_Number)
check_normality(lmm_Prop)
check_normality(lmm_Cov_num)
check_normality(lmm_Cov_dur)

# Check for homogeneity of variance

check_homogeneity(lmm_Number)
check_homogeneity(lmm_Prop)
check_homogeneity(lmm_Cov_num)
check_homogeneity(lmm_Cov_dur)

# Check for homoscedasticity

check_heteroscedasticity(lmm_Number)
check_heteroscedasticity(lmm_Prop)
check_heteroscedasticity(lmm_Cov_num)
check_heteroscedasticity(lmm_Cov_dur)

# Check for collinearity in linear mixed model

check_collinearity(lmm_Number)
check_collinearity(lmm_Prop)
check_collinearity(lmm_Cov_num)
check_collinearity(lmm_Cov_dur)
```

Analysis of the influence of social context on gaze to the confederate, assuming that data contains one value for the social and one for the non-social condition per participant

```
# T-test in case our data has a normal distribution
#First Look Latency
FL_Live <- filter(FirstLookLatency, Type.of.Instruction == 'Live')
FL_Video <- filter(FirstLookLatency, Type.of.Instruction == 'Video')
t.test(x = FL_Live$FirstLookLatency, y = FL_Video$FirstLookLatency,
       alternative = "two.sided", paired = FALSE,
       conf.level = 0.95)
cohensD(x = FL_Live$FirstLookLatency, y = FL_Video$FirstLookLatency) # compute effect size


wilcox.test(x = FL_Live$FirstLookLatency, y = FL_Video$FirstLookLatency,
       alternative = "two.sided", paired = FALSE,
       conf.level = 0.95)
FirstLookLatency %>% wilcox_effsize(FirstLookLatency ~ Type.of.Instruction) #compute effect size


#Proportion of Looks to the confederate
LP_Live <- filter(Proportion.Looks, Condition == 'Live')
LP_Video <- filter(Proportion.Looks, Condition == 'Video')
t.test(x = LP_Live$Proportion.Looks, y = LP_Video$Proportion.Looks,
       alternative = "two.sided", paired = FALSE,
       conf.level = 0.95)
cohensD(x = LP_Live$Proportion.Looks, y = LP_Video$Proportion.Looks) # compute effect size


wilcox.test(x = LP_Live$Proportion.Looks, y = LP_Video$Proportion.Looks,
       alternative = "two.sided", paired = FALSE,
       conf.level = 0.95)
Proportion.Looks %>% wilcox_effsize(Proportion.Looks ~ Condition)#compute effect size


#Number of direct looks to the confederate
NL_Live <- filter(Number.Looks, Condition == 'Live')
NL_Video <- filter(Number.Looks, Condition == 'Video')
t.test(x = NL_Live$Number.Looks, y = NL_Video$Number.Looks,
       alternative = "two.sided", paired = FALSE,
       conf.level = 0.95)
cohensD(x = NL_Live$Number.Looks, y = NL_Video$Number.Looks) # compute effect size

wilcox.test(x = NL_Live$Number.Looks, y = NL_Video$Number.Looks,
       alternative = "two.sided", paired = FALSE,
       conf.level = 0.95)
Number.Looks %>% wilcox_effsize(Number.Looks ~ Condition) #compute effect size
```

Analysis for gaze to the confederate (mixed ANOVA)

Note that such an ANOVA will be conducted for 3 different gaze variables (i.e. proportional looking duration, number of looks, and latency of the first look)

```
# If the model assumptions are not violated

#First Look Latency
bwtrim(FirstLookLatency~Type.of.Instruction*Emotion.Displayed, id = Participant.ID, FirstLookLatency)
#Pair-wise comparison with p adjusted
FirstLookLatency %>% group_by(Type.of.Instruction) %>% pairwise_t_test(FirstLookLatency ~ Emotion.Displayed, p.adjust.method = "bonferroni")

#Proportion of Looks to the confederate
anova_test(data = Proportion.Looks, dv = Proportion.Looks, wid = Participant.ID, within = Emotion.Displayed, between = Condition)
#Pair-wise comparison with p adjusted
Proportion.Looks %>% group_by(Condition) %>% pairwise_t_test(Proportion.Looks ~ Emotion.Displayed, p.adjust.method = "bonferroni")

bwtrim(Proportion.Looks~Condition*Emotion.Displayed, id = Participant.ID, Proportion.Looks)


#Number of direct looks to the confederate
anova_test(data = Number.Looks, dv = Number.Looks, wid = Participant.ID, within = Emotion.Displayed, between = Condition)
#Pair-wise comparison with p adjusted
Number.Looks %>% group_by(Condition) %>% pairwise_t_test(Number.Looks ~ Emotion.Displayed, p.adjust.method = "bonferroni")


bwtrim(Number.Looks~Condition*Emotion.Displayed, id = Participant.ID, Number.Looks)
```

Analysis for the effects of Sociality conditions and Expression on covert attention measures (mixed ANOVA)

Note that such an ANOVA will be conducted for 2 different gaze variables (i.e. looking duration and number of looks)

```
#Number of covert looks to the confederate
anova_test(data = Covert.Number, dv = Number.Looks, wid = Participant.ID, within = Emotion.Displayed, between = Condition)

#Pair-wise comparison with p adjusted
Covert.Number %>% group_by(Condition) %>% pairwise_t_test(Number.Looks ~ Emotion.Displayed, p.adjust.method = "bonferroni")

bwtrim(Number.Looks~Condition*Emotion.Displayed, id = Participant.ID, Covert.Number)

#Duration of covert looks to the confederate
anova_test(data = Covert.Duration, dv = Duration.Looks, wid = Participant.ID, within = Emotion.Displayed, between
= Condition)

#Pair-wise comparison with p adjusted
Covert.Duration %>% group_by(Condition) %>% pairwise_t_test(Duration.Looks ~ Emotion.Displayed, p.adjust.method =
"bonferroni")

bwtrim(Duration.Looks~Condition*Emotion.Displayed, id = Participant.ID, Covert.Duration)
```

Analysis of the effect of individual traits on gaze patterns (linear mixed model)

```
#Open data from questionnaires
Quest_data <- read.csv2(file = 'Participants_QuestionnaireScores.csv', sep=";", header=TRUE)
Quest_data <- Quest_data %>% mutate(Participant.ID = as.factor(Participant.ID)) %>% mutate(Type.of.Instruction =
as.factor(Type.of.Instruction)) %>% arrange(Participant.ID) %>% select(1:4)

# Bind to other data
Proportion.Looks %>% left_join(Quest_data, by = "Participant.ID") -> Proportion.Looks2
Number.Looks %>% left_join(Quest_data, by = "Participant.ID") -> Number.Looks2
Covert.Duration %>% left_join(Quest_data, by = "Participant.ID") -> Covert.Duration2
Covert.Number %>% left_join(Quest_data, by = "Participant.ID") -> Covert.Number2

# Subjects as random effects

# Proportion of Looks

model_base = rlmer(Proportion.Looks ~ Condition*Emotion.Displayed + (1|Participant.ID), data = Proportion.Looks2)
#effect of the interaction between sociality and expression as predictors
model_parameters(model_base)

model_AQ = rlmer(Proportion.Looks ~ Condition*Emotion.Displayed + AQ + (1|Participant.ID), data = Proportion.Looks2) # add effect of AQ
model_parameters(model_AQ)

model_SIAS = rlmer(Proportion.Looks ~ Condition*Emotion.Displayed + SIAS + (1|Participant.ID), data = Proportion.Looks2) # add effect of SIAS
model_parameters(model_SIAS)

model_AQ_SIAS = rlmer(Proportion.Looks ~ Condition*Emotion.Displayed + AQ + SIAS + (1|Participant.ID), data = Proportion.Looks2) # add both the effects of AQ and
model_parameters(model_AQ_SIAS)

#Comparing models
compare_performance(model_base, model_SIAS, rank = T) # Compare base model with the model adding SIAS as a predictor
compare_performance(model_base, model_AQ, rank = T) # Compare base model with the model adding AQ as a predictor
compare_performance(model_SIAS, model_AQ_SIAS, rank = T) #Compare model including only SIAS, with model including
both AQ and SIAS as fixed effects
compare_performance(model_AQ, model_AQ_SIAS, rank = T) #Compare model including only AQ, with model including both AQ and SIAS as fixed effects
compare_performance(model_base, model_AQ_SIAS, rank = T) # Compare model excluding AQ and SIAS, with model including both

#Number of Looks

model_base = rlmer(Number.Looks ~ Condition*Emotion.Displayed + (1|Participant.ID), data = Number.Looks2) #effect
of the interaction between sociality and expression as predictors
model_parameters(model_base)

model_AQ = rlmer(Number.Looks ~ Condition*Emotion.Displayed + AQ + (1|Participant.ID), data = Number.Looks2) # add effect of AQ
model_parameters(model_AQ)

model_SIAS = rlmer(Number.Looks ~ Condition*Emotion.Displayed + SIAS + (1|Participant.ID), data = Number.Looks2)
# add effect of SIAS
model_parameters(model_SIAS)
library("sjPlot")
plot_model(model_SIAS, type = "int")

model_AQ_SIAS = rlmer(Number.Looks ~ Condition*Emotion.Displayed + AQ + SIAS + (1|Participant.ID), data = Number.Looks2) # add both the effects of AQ and
model_parameters(model_AQ_SIAS)

#Comparing models
compare_performance(model_base, model_SIAS, rank = T) # Compare base model with the model adding SIAS as a predictor
compare_performance(model_base, model_AQ, rank = T) # Compare base model with the model adding AQ as a predictor
compare_performance(model_SIAS, model_AQ_SIAS, rank = T) #Compare model including only SIAS, with model including
both AQ and SIAS as fixed effects
compare_performance(model_AQ, model_AQ_SIAS, rank = T) #Compare model including only AQ, with model including both AQ and SIAS as fixed effects
compare_performance(model_base, model_AQ_SIAS, rank = T) # Compare model excluding AQ and SIAS, with model including both

# Duration of Covert looks

model_base = rlmer(Duration.Looks ~ Condition*Emotion.Displayed + (1|Participant.ID), data = Covert.Duration2) #effect of the interaction between sociality and expression as predictors
model_parameters(model_base)

model_AQ = rlmer(Duration.Looks ~ Condition*Emotion.Displayed + AQ + (1|Participant.ID), data = Covert.Duration2)
# add effect of AQ
model_parameters(model_AQ)

model_SIAS = rlmer(Duration.Looks ~ Condition*Emotion.Displayed + SIAS + (1|Participant.ID), data = Covert.Duration2) # add effect of SIAS
model_parameters(model_SIAS)

model_AQ_SIAS = rlmer(Duration.Looks ~ Condition*Emotion.Displayed + AQ + SIAS + (1|Participant.ID), data = Covert.Duration2) # add both the effects of AQ and
model_parameters(model_AQ_SIAS)

#Comparing models
compare_performance(model_base, model_SIAS, rank = T) # Compare base model with the model adding SIAS as a predictor
compare_performance(model_base, model_AQ, rank = T) # Compare base model with the model adding AQ as a predictor
compare_performance(model_SIAS, model_AQ_SIAS, rank = T) #Compare model including only SIAS, with model including
both AQ and SIAS as fixed effects
compare_performance(model_AQ, model_AQ_SIAS, rank = T) #Compare model including only AQ, with model including both AQ and SIAS as fixed effects
compare_performance(model_base, model_AQ_SIAS, rank = T) # Compare model excluding AQ and SIAS, with model including both

# Number of Covert Looks

model_base = rlmer(Number.Looks ~ Condition*Emotion.Displayed + (1|Participant.ID), data = Covert.Number2) #effect of the interaction between sociality and expression as predictors
model_parameters(model_base)

model_AQ = rlmer(Number.Looks ~ Condition*Emotion.Displayed + AQ + (1|Participant.ID), data = Covert.Number2) # add effect of AQ
model_parameters(model_AQ)

model_SIAS = rlmer(Number.Looks ~ Condition*Emotion.Displayed + SIAS + (1|Participant.ID), data = Covert.Number2)
# add effect of SIAS
model_parameters(model_SIAS)

model_AQ_SIAS = rlmer(Number.Looks ~ Condition*Emotion.Displayed + AQ + SIAS + (1|Participant.ID), data = Covert.Number2) # add both the effects of AQ and
model_parameters(model_AQ_SIAS)

#Comparing models
compare_performance(model_base, model_SIAS, rank = T) # Compare base model with the model adding SIAS as a predictor
compare_performance(model_base, model_AQ, rank = T) # Compare base model with the model adding AQ as a predictor
compare_performance(model_SIAS, model_AQ_SIAS, rank = T) #Compare model including only SIAS, with model including
both AQ and SIAS as fixed effects
compare_performance(model_AQ, model_AQ_SIAS, rank = T) #Compare model including only AQ, with model including both AQ and SIAS as fixed effects
compare_performance(model_base, model_AQ_SIAS, rank = T) # Compare model excluding AQ and SIAS, with model including both
```

Gaze-following occurences

```
#Open data from proportion of gaze following
GazeFollow <- read.csv2(file = 'GazeData.csv', sep=",", header=TRUE)
GazeFollow <- GazeFollow[ -c(5:10) ]
GazeFollow <- GazeFollow %>% mutate(Proportion.Gaze = as.numeric(Proportion.Gaze))
GazeFollow$Proportion.Gaze <- round(GazeFollow$Proportion.Gaze, digits=5)
#Table 1 - Gaze Following to the clipboard
formattable(GazeFollow, align =c("l","l","c","c", "c"),
            list(`Indicator Name` = formatter(
                "span", style = ~ style(color = "grey",font.weight = "bold"))
            ))
```

Participant.ID	Emotion.Displayed	Number.Looks	Condition	Proportion.Gaze
LP11	Negative	1	Live	0.00058
LP19	Neutral	1	Live	0.00055
VP02	Positive	2	Video	0.00119
VP03	Neutral	1	Video	0.00057
VP04	Negative	1	Video	0.00057
VP05	Neutral	1	Video	0.00056
VP10	Neutral	1	Video	0.00060
VP10	Positive	3	Video	0.00190
VP11	Negative	1	Video	0.00056
VP20	Neutral	1	Video	0.00059

```
#Open data from looks to the clipboard
GazeClipboard <- read.csv2(file = 'GazeClipboard.csv', sep=",", header=TRUE)
GazeClipboard <- GazeClipboard %>% spread(key = Emotion.Displayed, value = Number.Looks) %>% replace(is.na(.), 0)
#Table 2 - Number of looks to the clipboard
formattable(GazeClipboard, align =c("l","l","c","c", "c"),
            list(`Indicator Name` = formatter(
                "span", style = ~ style(color = "grey",font.weight = "bold"))
            ))
```

Participant.ID	Condition	Negative	Neutral	Positive
LP01	Live	0	0	1
LP02	Live	1	0	0
LP03	Live	1	0	0
LP04	Live	0	1	0
LP05	Live	0	1	0
LP06	Live	1	0	0
LP11	Live	1	0	2
LP18	Live	1	0	1
LP19	Live	0	1	1
LP20	Live	1	0	0
LP21	Live	1	0	0
LP22	Live	1	0	0
LP24	Live	0	0	1
VP01	Video	5	2	1
VP02	Video	8	7	7
VP03	Video	0	7	2
VP04	Video	3	4	0
VP05	Video	0	1	0
VP06	Video	0	3	4
VP08	Video	2	3	0
VP09	Video	2	3	0
VP10	Video	9	7	24
VP11	Video	1	2	2
VP12	Video	10	3	6
VP15	Video	12	13	7
VP17	Video	2	5	0
VP18	Video	0	1	0
VP19	Video	17	21	3
VP20	Video	3	5	0
VP21	Video	1	0	0
VP22	Video	1	0	0
VP24	Video	7	1	1

Plotting graphs

```
# Proportion of Looks
data_plot = summarySE(data = Proportion.Looks, measurevar="Proportion.Looks", groupvars=c("Condition","Emotion.Displayed"))
ggplot(data_plot, aes(Emotion.Displayed, Proportion.Looks, fill=Emotion.Displayed)) +
  facet_grid(cols = vars(Condition)) +
  geom_bar(stat="identity", position="dodge") + theme_minimal() +
  geom_errorbar(aes(x = Emotion.Displayed, ymin=Proportion.Looks-se, ymax=Proportion.Looks+se), width=.2,
              position=position_dodge(.9)) +
  scale_fill_manual(values=c('gray48','gray76','gray27'),
                    name = "Condition") +
  labs(x="Expression Displayed", y = "Proportion of Looking Time") +
  scale_x_discrete(labels = c('Negative','Neutral','Positive')) +
  theme(legend.title=element_blank(), text = element_text(size = 13),
        axis.title = element_text(size = 13), legend.position = 'none')

# Number of Looks
data_plot2 = summarySE(data = Number.Looks, measurevar="Number.Looks", groupvars=c("Condition","Emotion.Displayed"))
ggplot(data_plot2, aes(Emotion.Displayed, Number.Looks, fill=Emotion.Displayed)) +
  facet_grid(cols = vars(Condition)) +
  geom_bar(stat="identity", position="dodge") + theme_minimal() +
  geom_errorbar(aes(x = Emotion.Displayed, ymin=Number.Looks-se, ymax=Number.Looks+se), width=.2,
              position=position_dodge(.9)) +
  scale_fill_manual(values=c('gray48','gray76','gray27'),
                    name = "Condition") +
  labs(x="Expression Displayed", y = "Number of Looks") +
  scale_x_discrete(labels = c('Negative','Neutral','Positive')) +
  theme(legend.title=element_blank(), text = element_text(size = 13),
        axis.title = element_text(size = 13), legend.position = 'none')

# Number of Covert looks
data_plot3 = summarySE(data = Covert.Number, measurevar="Number.Looks", groupvars=c("Condition","Emotion.Displayed"))
ggplot(data_plot3, aes(Emotion.Displayed, Number.Looks, fill=Emotion.Displayed)) +
  facet_grid(cols = vars(Condition)) +
  geom_bar(stat="identity", position="dodge") + theme_minimal() +
  geom_errorbar(aes(x = Emotion.Displayed, ymin=Number.Looks-se, ymax=Number.Looks+se), width=.2,
              position=position_dodge(.9)) +
  scale_fill_manual(values=c('gray48','gray76','gray27'),
                    name = "Condition") +
  labs(x="Expression Displayed", y = "Number of Covert Looks") +
  scale_x_discrete(labels = c('Negative','Neutral','Positive')) +
  theme(legend.title=element_blank(), text = element_text(size = 13),
        axis.title = element_text(size = 13), legend.position = 'none')

# Duration of Covert looks
data_plot4 = summarySE(data = Covert.Duration, measurevar="Duration.Looks", groupvars=c("Condition","Emotion.Displayed"))
ggplot(data_plot4, aes(Emotion.Displayed, Duration.Looks, fill=Emotion.Displayed)) +
  facet_grid(cols = vars(Condition)) +
  geom_bar(stat="identity", position="dodge") + theme_minimal() +
  geom_errorbar(aes(x = Emotion.Displayed, ymin=Duration.Looks-se, ymax=Duration.Looks+se), width=.2,
              position=position_dodge(.9)) +
  scale_fill_manual(values=c('gray48','gray76','gray27'),
                    name = "Condition") +
  labs(x="Expression Displayed", y = "Duration of Covert Looks to Confederate") +
  scale_x_discrete(labels = c('Negative','Neutral','Positive')) +
  theme(legend.title=element_blank(), text = element_text(size = 13),
        axis.title = element_text(size = 13), legend.position = 'none')
```

```
report::cite_packages()
```


```
##   - ##############################################################################The main WRS2 article:
##   - H. Wickham. ggplot2: Elegant Graphics for Data Analysis. Springer-Verlag New York, 2016.
##   - Hadley Wickham (2011). The Split-Apply-Combine Strategy for Data Analysis. Journal of Statistical Software, 40(1), 1-29. URL https://www.jstatsoft.org/v40/i01/.
##   - JJ Allaire and Yihui Xie and Jonathan McPherson and Javier Luraschi and Kevin Ushey and Aron Atkins and Hadley Wickham and Joe Cheng and Winston Chang and Richard Iannone (2022). rmarkdown: Dynamic Documents for R. R package version 2.14. URL https://rmarkdown.rstudio.com.
##   - Lüdecke D, Ben-Shachar M, Patil I, Makowski D (2020). "Extracting, Computing and Exploring the Parameters of Statistical Models using R." _Journal of Open Source Software_, *5*(53), 2445. doi:10.21105/joss.02445 <https://doi.org/10.21105/joss.02445>.
##   - Lüdecke et al., (2021). performance: An R Package for Assessment, Comparison and Testing of Statistical Models. Journal of Open Source Software, 6(60), 3139. https://doi.org/10.21105/joss.03139
##   - Makowski, D., Ben-Shachar, M.S., Patil, I. & Lüdecke, D. (2020). Automated Results Reporting as a Practical Tool to Improve Reproducibility and Methodological Best Practices Adoption. CRAN. Available from https://github.com/easystats/report. doi: .
##   - Navarro, D. J. (2015) Learning statistics with R: A tutorial for psychology students and other beginners. (Version 0.6) University of New South Wales. Sydney, Australia
##   - R Core Team (2022). R: A language and environment for statistical computing. R Foundation for Statistical Computing, Vienna, Austria. URL https://www.R-project.org/.
##   - Sarkar, Deepayan (2008) Lattice: Multivariate Data Visualization with R. Springer, New York. ISBN 978-0-387-75968-5
##   - Wickham et al., (2019). Welcome to the tidyverse. Journal of Open Source Software, 4(43), 1686, https://doi.org/10.21105/joss.01686
##   - NA
##   - NA
##   - NA
##   - NA
##   - NA
##   - NA
##   - NA
##   - NA
##   - NA
##   - NA
##   - NA
##   - NA
##   - NA
##   - NA
##   - NA
```
